# Programmable Coacervates Based on Minimalist Sticker–Spacer Frameworks: Chemical Design, Functions, and Emerging Applications

**DOI:** 10.1021/acsami.6c03853

**Published:** 2026-04-22

**Authors:** Nadeem Essa, Manzar Abbas

**Affiliations:** † Department of Chemistry, 105955 Khalifa University of Science and Technology, Abu Dhabi, P.O. Box 127788, United Arab Emirates; ‡ Functional Biomaterials Group, Khalifa University of Science and Technology, Abu Dhabi, P.O. Box 127788, United Arab Emirates

**Keywords:** sticker-spacer frameworks, peptides, coacervates, protocells, biocatalysis, delivery systems

## Abstract

Membraneless compartments, such as coacervates, have emerged as powerful biomimetic models of protocells and fundamental regulators of the spatiotemporal organization of biochemical processes in living systems. The sticker–spacer framework offers a reductionist yet predictive approach for understanding and programming liquid–liquid phase separation (LLPS). In this review, we critically examine how the chemical identity of stickers and the architecture of spacers govern phase behavior, emphasizing that precise molecular design is often essential to achieve well-defined coacervate morphologies and to unravel their underlying physicochemical principles. We highlight recent advances in both simple and complex coacervate formed through sticker–spacer frameworks, physical and chemical properties of sticker and spacers, in situ spacer formation, light-responsive assemblies, and transition of coacervate droplets into thermodynamically stable nano assemblies. Key examples of chemical reactions within coacervates and delivery of drugs and macromolecules using sticker–spacer-engineered coacervates as carriers are discussed. Finally, we present an outlook that underscores the versatility of designer sticker–spacer frameworks as a unifying strategy for constructing adaptive coacervate protocells with broad potential in biocatalysis and biomedical applications.

## Graphical abstract

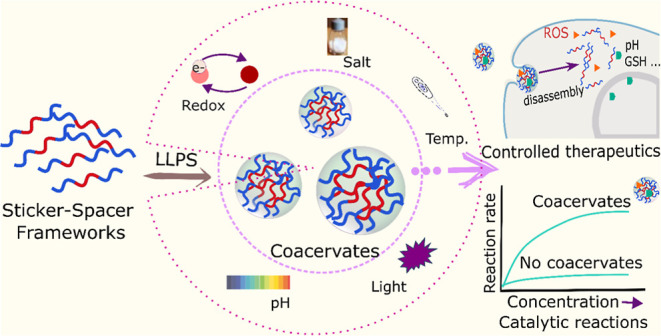

## 1 Introduction

Although the fundamental principles of biological evolution are well established, the chemical evolution that preceded the emergence of the first living cell remains a subject of active debate. This question continues to attract broad interest across diverse disciplines, including astrobiology, biophysics, prebiotic chemistry, and synthetic biology.^1–4^ A defining hallmark of life is compartmentalization, which is widely believed to have played a crucial role in the formation of the earliest cells. By spatially localizing reactions within segregated environments, compartmentalization enables effective coordination of complex biochemical networks.^5–8^ Such compartments can enhance reaction rates or, conversely, suppress them, depending on their chemical composition and the local physicochemical conditions they impose.^9,10^

Lipid vesicles are widely regarded as plausible primitive compartments because of the close resemblance between their lipid bilayer membranes and those of modern living cells.^11–13^ Similarly, amphiphilic polymersomes have been extensively explored as protocell models due to their ability to self-assemble into vesicular architectures.^14,15^ However, membrane-bound compartments often exhibit limited permeability, which may restrict the exchange of biomolecular precursors thought to be essential under prebiotic conditions.^16,17^ These limitations have motivated the search for alternative compartmentalization strategies that permit dynamic molecular exchange without reliance on membranes.

In this context, increasing attention has been directed toward membraneless organelles (MLOs), such as Stress granules (SGs), P-bodies, and nucleoli.^18,19^ These cellular compartments are primarily composed of intrinsically disordered proteins (IDPs), often in association with other biomolecules such as nucleic acids.^20–23^ Their assembly is governed by LLPS, a spontaneous and reversible process that gives rise to dynamic, condensed phases within the cellular environment, along with a coexisting dilute phase that is depleted of solutes. Inspired by the physicochemical principles underlying MLO formation, coacervates, often referred to as artificial condensates, have emerged as a promising class of materials for a wide range of applications, including drug delivery and biocatalysis.^24–27^

Owing to their life-like properties, coacervates are widely regarded as protocell models with the potential to elucidate chemically regulated mechanisms that may have operated during prebiotic evolution.^28^ Their inherent ability to sequester guest molecules and spatially localize reactants has motivated extensive research into the design and characterization of synthetic coacervate systems. Coacervates can be broadly classified into two categories: (a) heterotypic coacervates and (b) homotypic coacervates. Heterotypic coacervates consist of two or more oppositely charged polyelectrolytes while homotypic coacervates, by comparison, arise from a single molecular species undergoing phase separation through self-associative interactions.^29,30^ One of the key advantages of coacervates is their pronounced response to external stimuli. Coacervate droplets can undergo reversible mixing and demixing in response to environmental parameters such as pH, salt concentration, and temperature.^31–35^ Despite these favorable attributes, coacervates also exhibit notable limitations. Their ultralow interfacial tension can result in droplet instability and coalescence, while their often complex, multicomponent compositions may limit their broader applicability as simplified and controllable models for artificial cells. Many of the well-studied complex coacervates may also suffer from low biocompatibility because of incomplete purification of the polymeric residue. Hence, the use of biomolecule-based coacervates is extensively being employed for biological contexts.

To address these challenges, peptide-based coacervates have emerged as particularly attractive candidates, owing to their structural simplicity, biocompatibility, and precise sequence-controlled tunability.^28^ In addition, peptides can incorporate chemically functional side chains or catalytic moieties that selectively enrich reactants and, in some cases, lower reaction activation barriers as well as having the inherent ability of being responsive to physiological stimuli.^36^ Side chain functionality and their respective p*K*_a_ shifts are also taken into account when determining conditions under which phase separation occurs, such as pH and ionic strength. The versatility of peptides arises from the wide range of amino acid side-chain functionalities available, encompassing hydrophilic, hydrophobic, and charged groups. The interplay among these functionalities governs peptide conformation, or, in some cases, intrinsic disorder, and ultimately dictates phase-separation behavior.^37^

Importantly, recent studies have demonstrated that even minimal peptide motifs, such as dipeptides and tripeptides, are capable of undergoing phase separation.^38,39^ These reviews highlight the utility of peptide-based model systems for elucidating the fundamental mechanisms governing phase separation in both membraneless organelles and synthetic coacervates. Although considerable efforts have been devoted to identifying the suitable building blocks and empirical design rules for coacervation, a notable gap remains in the development of simplified experimental models that could possess the life-mimicking properties in these systems. Therefore, in recent years, the sticker–spacer framework has emerged as a potent strategy for improving the predictability of phase-separating behavior. By adoption of this approach, peptide-based coacervates can be rationally designed using defined sequences that enable tunable physicochemical properties and support a broad range of applications.

In this review, we critically evaluate recent studies demonstrating the effectiveness of sticker–spacer designer frameworks for coacervate formation. We first introduce the fundamental principles of the sticker–spacer frameworks and highlight its relevance for understanding coacervate formation and phase separation behavior. We then discuss the design principles inspired by intrinsic molecular properties, including in situ formation and light responsiveness in dynamic environments. Subsequently, we consolidate and analyze several important chemical reactions occurring within coacervates, with particular emphasis on their thermodynamic and kinetic parameters. This focus on nonenzymatic regimes addresses a gap in the current literature, which has predominantly emphasized enzymatic reactions. Finally, we highlight the potential of sticker–spacer-based coacervates as versatile biomaterials for applications in biocatalysis and delivery carriers for small (therapeutic agents) and large (nucleotides) molecules.

## 2 Rational Design of Sticker-Spacer Compounds and Driving Forces

Since the discovery of MLOs, extensive research has focused on elucidating their structural organization and their potent role in LLPS-mediated cellular processes. Coacervates formed via LLPS are typically formed from relatively large biomolecules, including IDPs, polymers, nucleotides, and polypeptides. The widely used approach to investigate LLPS involves identifying low-complexity regions (LCRs) within proteins, which lack a stable three-dimensional conformation and are key contributors to phase separation.^40,41^ However, these LCRs often constitute large and heterogeneous macromolecular domains, making it difficult to isolate the specific molecular features that govern LLPS.^23^ Remarkably, many phase-separating proteins contain prion-like domains, enriched in polar amino acids and interspersed with aromatic residues, which are thought to mediate the multivalent interactions underlying phase separation.^42–44^ This recurring sequence architecture suggests that specific residue patterning rather than overall molecular size alone plays a decisive role in LLPS.

Motivated by this insight, Martin and co-workers developed the sticker–spacer framework using combined experimental and computational approaches to rationalize phase-separation behavior.^45^ In this model, aromatic residues act as associative stickers, while intervening polar sequences function as flexible spacers, together governing interaction valency, temperature-dependent assembly, and suppression of irreversible aggregation. This reductionist framework has proven highly effective in distilling the complex physicochemical origins of LLPS into actionable design principles and has enabled bottom-up strategies for engineering phase-separating systems.

Building on this concept, we demonstrated that short peptide derivatives can be rationally engineered to undergo reversible coacervation while avoiding irreversible aggregation.^46^ Specifically, insertion of a cystamine spacer between two diphenylalanine (FF) motifs yielded FFssFF. Whereas diphenylalanine alone exhibits poor aqueous solubility and a strong tendency toward irreversible aggregation,^47^ the introduction of a single spacer markedly enhanced solubility under mildly acidic conditions, enabling complete molecular mixing prior to phase separation. Upon increasing the pH, the system underwent spontaneous coacervation, forming micrometer-sized droplets as confirmed by turbidity measurements and optical microscopy. This example highlights how minimal molecular modifications can precisely tune solubility and reversibility, underscoring the power of reductionist design before it advances to larger and more complex protocell architectures.

By integrating experimental and computational approaches,^27^ this model enables the extraction of systematic design principles governing phase behavior, offering both mechanistic insight and practical guidance for the rational engineering of dynamic condensates. Beyond intrinsic sequence features, ligand-mediated phase separation represents a natural extension of the sticker–spacer framework. In this context, ligands can function as pseudostickers or pseudospacers, either promoting or suppressing phase separation depending on their valency and binding preferences. Coarse-grained simulations by Ruff and co-workers revealed that monovalent ligands generally inhibit phase separation by occupying sticker sites or increasing effective spacer volume, thereby reducing interaction valency.^48^ In contrast, divalent ligands may either enhance or suppress condensation, depending on whether they facilitate interchain cross-linking or compete with native sticker–sticker interactions. These findings illustrate how external regulators can modulate phase behavior even below intrinsic saturation concentrations.

Importantly, the sticker–spacer framework has also proven to be highly informative in mutation-based studies with direct relevance to disease. Dysregulation of biomolecular condensates is implicated in several neurodegenerative disorders, including amyotrophic lateral sclerosis. Yang and co-workers investigated the LLPS behavior of a segment of the UBQLN2 protein and identified discrete sticker regions critical for condensation.^49^ The systematic substitution of sticker and spacer residues could reveal that even single–amino acid changes can dramatically alter phase behavior, including shifts in temperature-dependent phase boundaries and droplet dynamics. Importantly, the replacement of native sticker residues with aromatic amino acids has enhanced phase separation at substantially lower temperatures, highlighting the exquisite sensitivity of LLPS to local sequence chemistry and reinforcing the predictive power of the sticker–spacer frameworks at the single-residue level.

LLPS which is the main driving force to form coacervate droplets from sticker-spacer frameworks can be broadly classified into three types: (1) associative, (2) segregative, and (3) simple phase separation (Figure 1a). In associative phase separation, two water-soluble molecules partition into the same condensed phase due to mutually attractive interactions. The resulting dense phase, termed a coacervate, is enriched in both components and typically contains a substantial amount of solvent (often more than 50 wt %). The coexisting dilute phase is depleted of both solutes and consists predominantly of solvent. The classical example of associative LLPS is the phase separation between two oppositely charged polymers, where electrostatic attraction acts as the primary driving force. In addition to electrostatics, hydrogen bonding, cation–π interactions, π–π stacking, and other multivalent weak interactions can collectively stabilize the coacervate phase. In contrast, segregative phase separation could arise from mutually repulsive interactions between the two soluble components, like sticker-spacer frameworks, polymers, or nucleotides. Because of unfavorable interactions between them, the components demix into two distinct phases, each enriched in one species. Here, phase separation is driven by incompatibility, often described in terms of unfavorable enthalpic interactions or excluded-volume effects, rather than attractive association.^50^ The coexistence of phases and the conditions under which phase separation occurs are typically represented by using a phase diagram (Figure 1b). The third type, simple phase separation, involves a single component that undergoes self-association. In this case, attractive interactions among identical molecules become sufficiently strong under specific conditions (e.g., temperature, pH, concentration, or ionic strength) to overcome solvation effects, rendering the molecule partially insoluble and leading to the formation of a condensed phase. This dense phase, referred to as a simple coacervate, shares many physicochemical characteristics with complex coacervates formed through associative phase separation. The sticker spacer frameworks frequently exhibit simple and complex coacervation to form membraneless organelles like droplets driven by multivalent interactions such as charge–charge attraction, cation–π interactions, and π–π stacking (Figure 1c).

**Figure 1:**
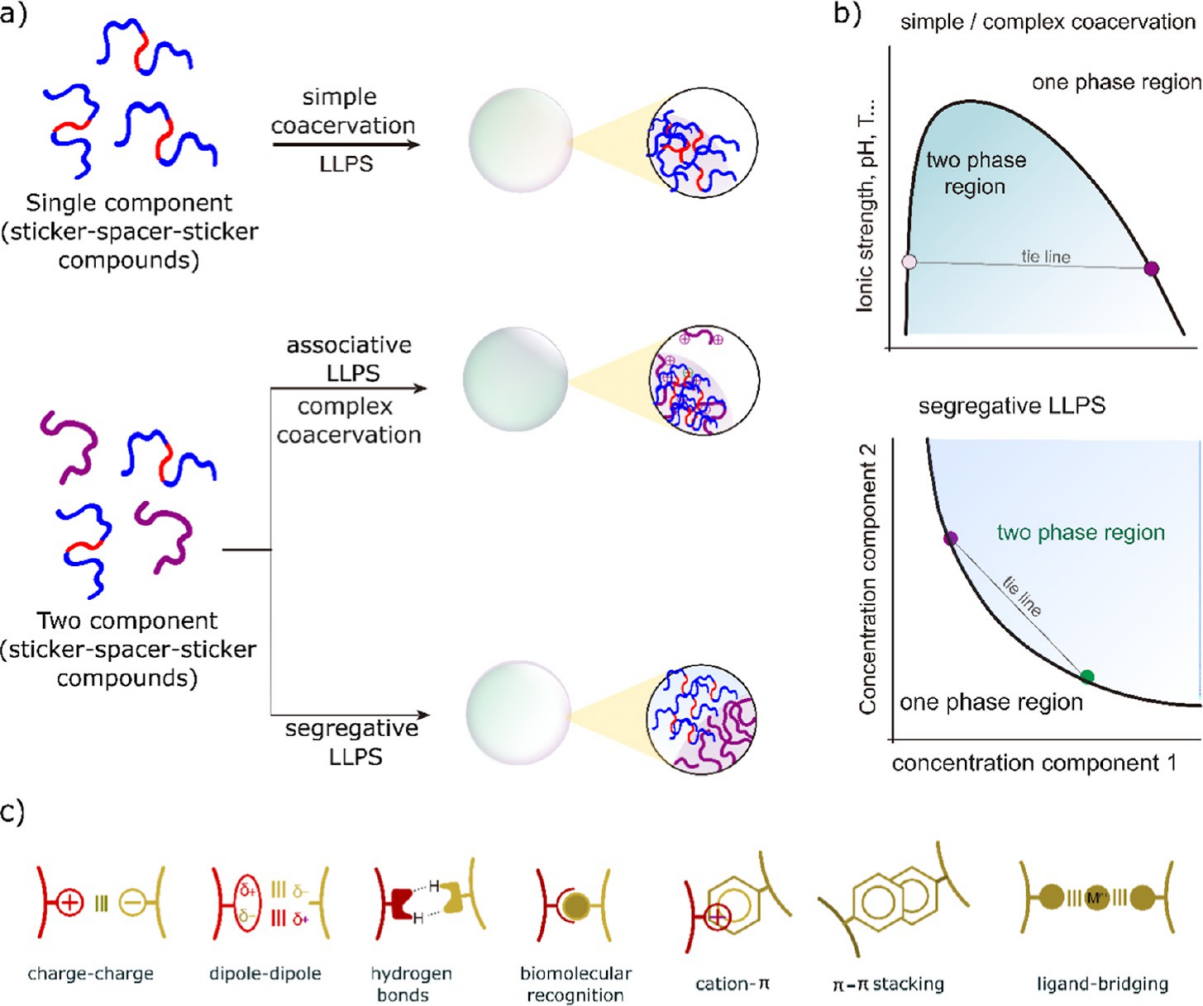
Schematic illustration of sticker-spacer-based frameworks for phase separation and driving forces, (a) role of sticker-spacer-stickers frameworks for simple and complex coacervation, (b) phase diagrams, (c) different noncovalent molecular interactions involved in the formation of coacervates. Reproduced from ref 28 available under a CC-BY-NC license. Copyright 2021.

From a theoretical point of view, simple coacervation is often described using Flory–Huggin’s theory, which models phase separation in terms of polymer–solvent mixing thermodynamics. In this framework, LLPS occurs when the free energy of mixing becomes unfavorable, leading to demixing into polymer-rich and polymer-poor phases. Although the molecular origins differ, simple and associative phase separation shares a common outcome: enrichment of macromolecules in a condensed phase stabilized by a balance of enthalpic interactions and entropic contributions. In general, the driving forces governing LLPS depend on whether interactions are predominantly attractive (associative and simple) or repulsive (segregative), but in all cases, the phase behavior emerges from the interplay between intermolecular interactions, solvent quality, and thermodynamic conditions.

## 3 Sticker-Spacer Designer Frameworks

The sticker–spacer frameworks provide a unifying and versatile platform for elucidating the systematic design principles that govern phase behavior, offering both mechanistic insight and practical guidance for the rational engineering of dynamic coacervate protocells. In this section, we discuss recent literature from the past five years that focuses on sticker–spacer-based architectures, in which stickers (Section 3.1) include peptides, aromatic moieties, and polypeptides, while spacers (Section 3.2 comprise non-natural linkers, cysteine residues, and peptide segments utilized both in simple and complex coacervates (Figure 2). Section 3.3 highlights the use of photoactive stickers for the construction of light-responsive coacervates, whereas Section 3.4 focuses on phase transitions of liquid-like droplets to solid-like nanofibrous assemblies.

**Figure 2:**
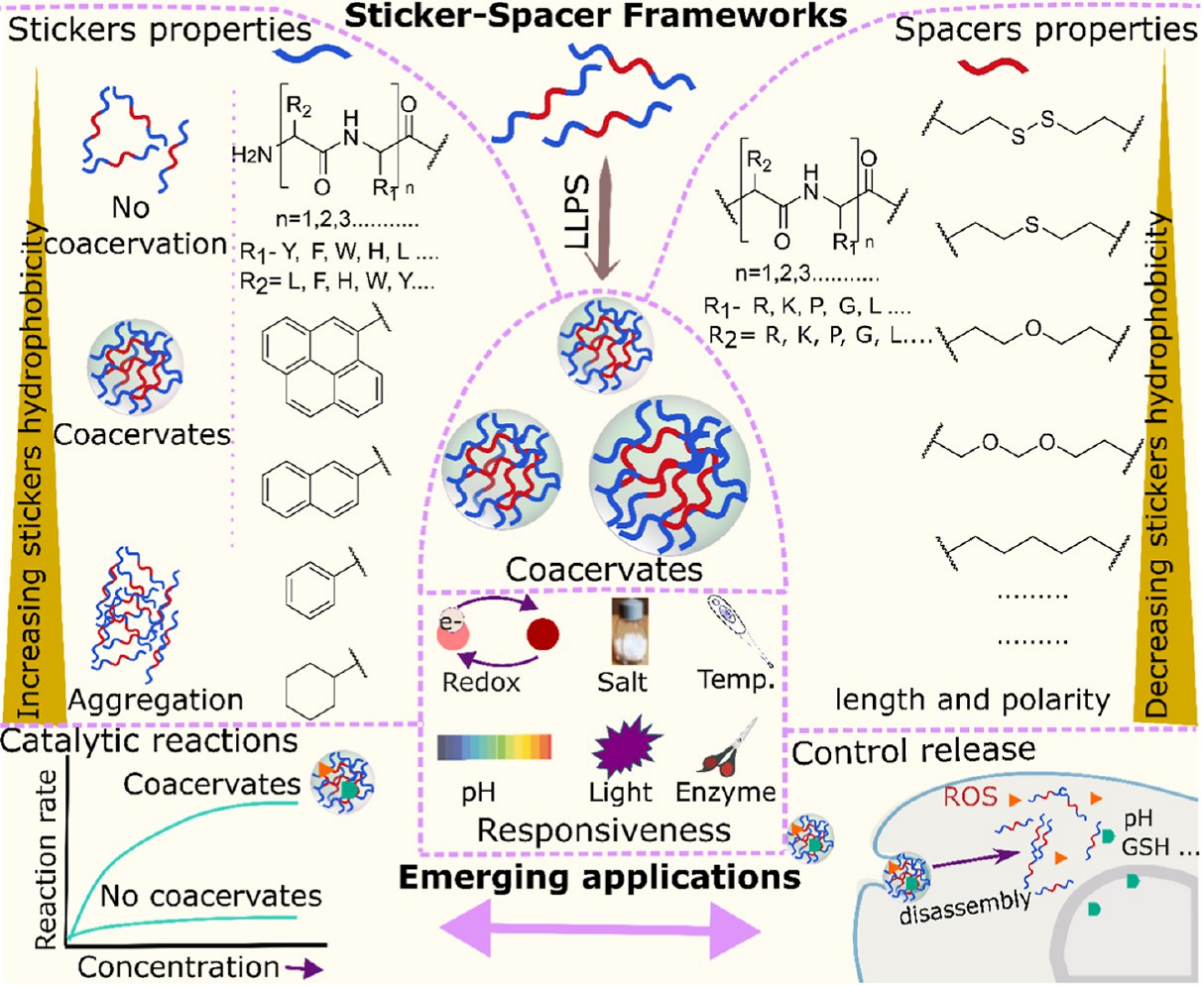
Schematic illustration to show the LLPS of sticker-spacer frameworks, stickers and spacer chemistry, coacervate functions, and emerging applications of sticker-spacer-based coacervates.

### 3.1 Stickers for Coacervation

The chemistry of sticker motifs plays a central role in tuning the physicochemical properties of coacervates at the interface of materials science. In the following sections, we discuss how hydrophobic and hydrophilic interactions govern LLPS and drive the formation, stability, and properties of coacervate assemblies.

#### 3.1.1 Dipeptide-Based Stickers for Coacervation

Dipeptides and their derivatives, particularly those containing aromatic residues, have been widely reported to self-assemble into nanostructures for diverse biotechnological applications.^51,52^ However, more recently, subtle chemical modifications based on the sticker-spacer framework have enabled dipeptides to participate in LLPS, leading to the formation of coacervates in which they play a central structural role.^38,39^ In parallel, these dipeptide coacervates were shown to exhibit characteristics that complement protocell models (Section 3.2).

Protocell research seeks to recapitulate simplified chemical systems that mimic the early processes underlying the emergence of the first living cells.^48,53^ A major challenge in this field is the complexity of integrating multiple defining features of life within a single, coordinated synthetic platform.^53^ Employing the commonly studied macromolecular complex coacervates for this endeavor, however, can potentially introduce another layer of complexity. In this context, reductionist strategies, such as the sticker–spacer framework, offer clear conceptual advantages.^30^ One promising approach begins with simple, low-molecular-weight building blocks capable of forming homotypic coacervates, followed by the stepwise introduction of functional complexity. Such complexity may arise through chemical transformations of the scaffolding material, environmental stimuli, prebiotically relevant reactions, or even through coencapsulation of other simple coacervates to produce biomimicking multiphasic structures. Unlike traditional coacervates, the sticker–spacer approach allows the systematic tuning of functionality through the rational placement of interacting motifs (stickers) and solubilizing segments (spacers), offering enhanced design flexibility. Numerous studies have therefore explored the strategies to impart protocell-like properties to coacervate droplets,^37,54,55^ bridging minimal chemical systems with emergent biological functionality.^30^

Among minimal building blocks, diphenylalanine (FF) represents a particularly well-studied example due to its strong propensity for self-assembly into nanoparticles or disordered aggregates. This behavior is primarily driven by π–π stacking between phenyl rings.^56,57^ FF also serves as a core recognition motif in polypeptides associated with neurodegenerative diseases. To modulate its physicochemical properties and assembly behavior across different solvent environments, FF has been extensively functionalized with non-natural moieties, thereby attenuating its intrinsic hydrophobicity and tuning its aggregation profile.^58^

Building on these insights and with the aim to harness LLPS-mediated assembly for coacervate formation, rationally designed stickers–cystamine–stickers were constructed by introducing a hydrophilic cystamine (disulfide) linker as the spacer between FF and several other dipeptides as stickers were synthesized.^46^ This modification enhances water solubility and allows coacervation to be readily triggered by a simple adjustment of the pH to 7.0 (Figure 3a). In contrast, when the same spacer was retained but the aromatic stickers were replaced with the nonaromatic dileucine (LL), coacervation was not observed under the same concentrations and conditions. Substitution of a single aromatic residue in stickers such as LF, restored coacervate formation, albeit at slightly elevated concentrations. Conversely, further increasing aromaticity through tryptophan-based stickers (WW or WF) promoted uncontrolled aggregation rather than well-defined condensates. This highlights that a delicate balance between aromatic/hydrophobic interactions and solubility of sticker motifs is required to induce the LLPS.

**Figure 3:**
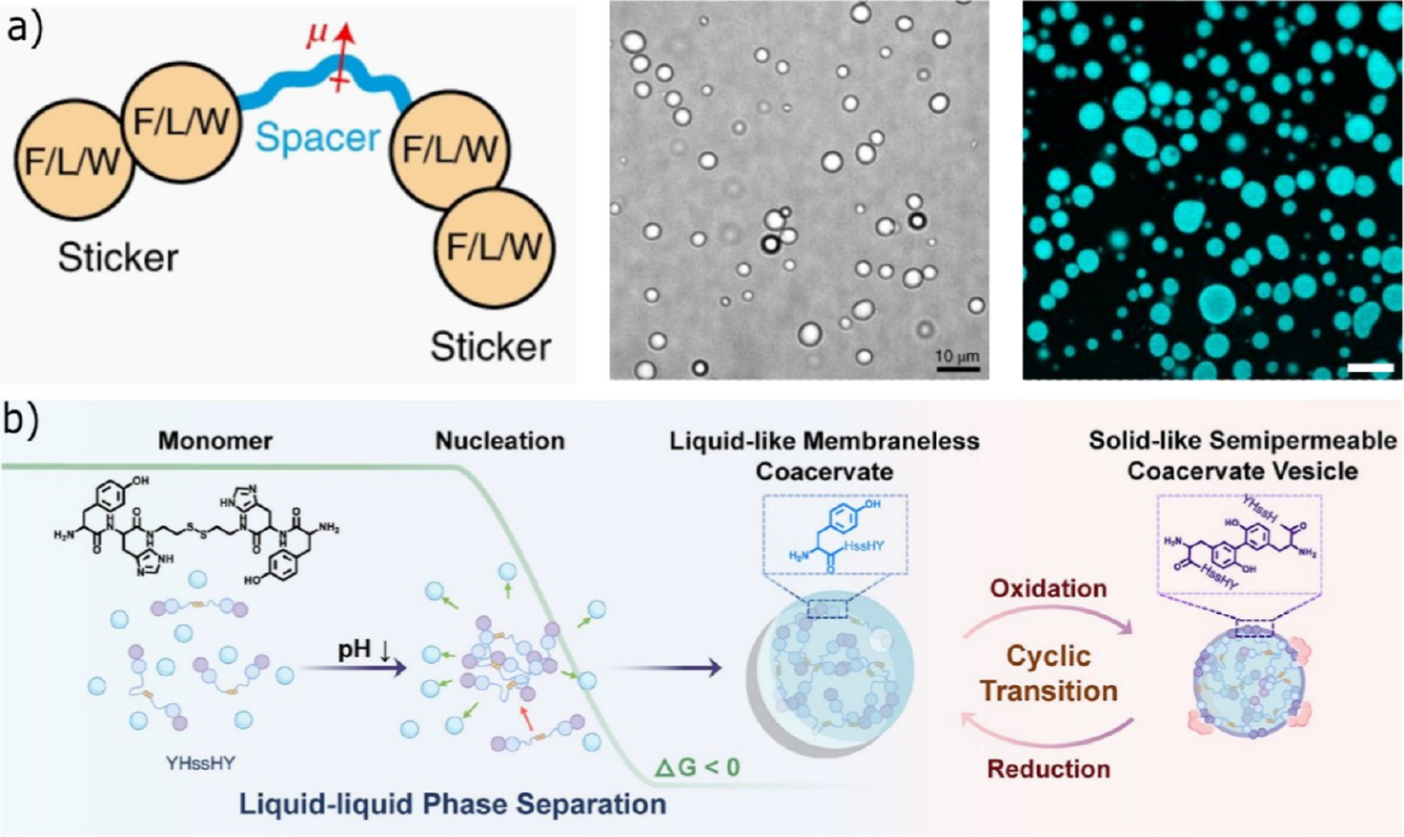
Chemical design and illustrations of sticker-spacer peptide derivatives, (a) schematic sticker-spacer library along with selected optical and fluorescent microscope images. Scale bar-10 μm. Reproduced from ref 46 with permission from @ copyright 2021, Nature Publishing Group, (b) schematic illustration to show formation and transformation of coacervates into coacervate vesicles. Reproduced with permission from ref 59 @ copyright 2026, John Wiley and Sons.

Coacervates formed from dipeptide stickers and hydrophilic spacers can also undergo nucleation into thermodynamically stable nanofiber architectures based on sticker hydrophobicity or a lack of spacer hydrophilicity. While this structural transition may be advantageous in certain contexts, it limits the utility of these compartments for applications that require dynamic, fluid-like behavior, such as protocell models.^60^ To address this limitation, dipeptide stickers can be tuned by introducing an amino acid with cross-linking ability that could potentially stabilize the droplets, such as a tyrosine residue. Tyrosine has a strong propensity to undergo oxidation, leading to the formation of dityrosine cross-links.^61^ By exploiting the chemistry of the YF dipeptide in combination with different spacers, we observed a transformation from coacervate droplets to coacervate-core vesicles that remained stable for several days. The resulting dityrosine-based membrane was semipermeable, intrinsically fluorescent, and mechanically flexible, highlighting its potential for biomimetic compartmentalization.^62^ Building on this dityrosine chemistry, Chen and co-workers further developed YH dipeptide stickers incorporating the same disulfide-containing spacer. This system enabled precise control over the redox-triggered transformation of coacervates into coacervate vesicles (Figure 3b), demonstrating a versatile strategy for regulating structural transitions in peptide-based phase-separated systems.^59^

Extending this design principle, Zhou and co-workers demonstrated that both coacervate formation and internal organization can be programmed by systematically varying amino acid composition in tryptophan-containing tetrapeptides, which show phase separation by changing the pH temperature.^63^ These peptides initially designed with a WXXW motif (X = any amino acid) exhibited relatively high saturation concentrations. Introduction of an additional tryptophan residue within the spacer region (WXWW) increases valency and consequently lowers the critical concentration for phase separation, which is consistent with multivalency principles. Besides modulating the phase behavior, these peptide sequences could provide the control over internal architecture of coacervates and different morphologies depending on amino acids used as spacers, ranging from stable coacervates, aggregates and no coacervation (Figure 4a–c). The fluorophore encapsulation experiments revealed a pronounced dependence of morphology on the order of guest addition: fluorophores introduced after coacervate formation formed the vesicular structures, whereas fluorophores present prior to phase separation yielded core–shell architectures through π–π stacking. These findings suggest that the timing of molecular uptake can significantly influence the internal organization and provide insights into plausible primitive compartmentalization mechanisms for cellular life.

**Figure 4:**
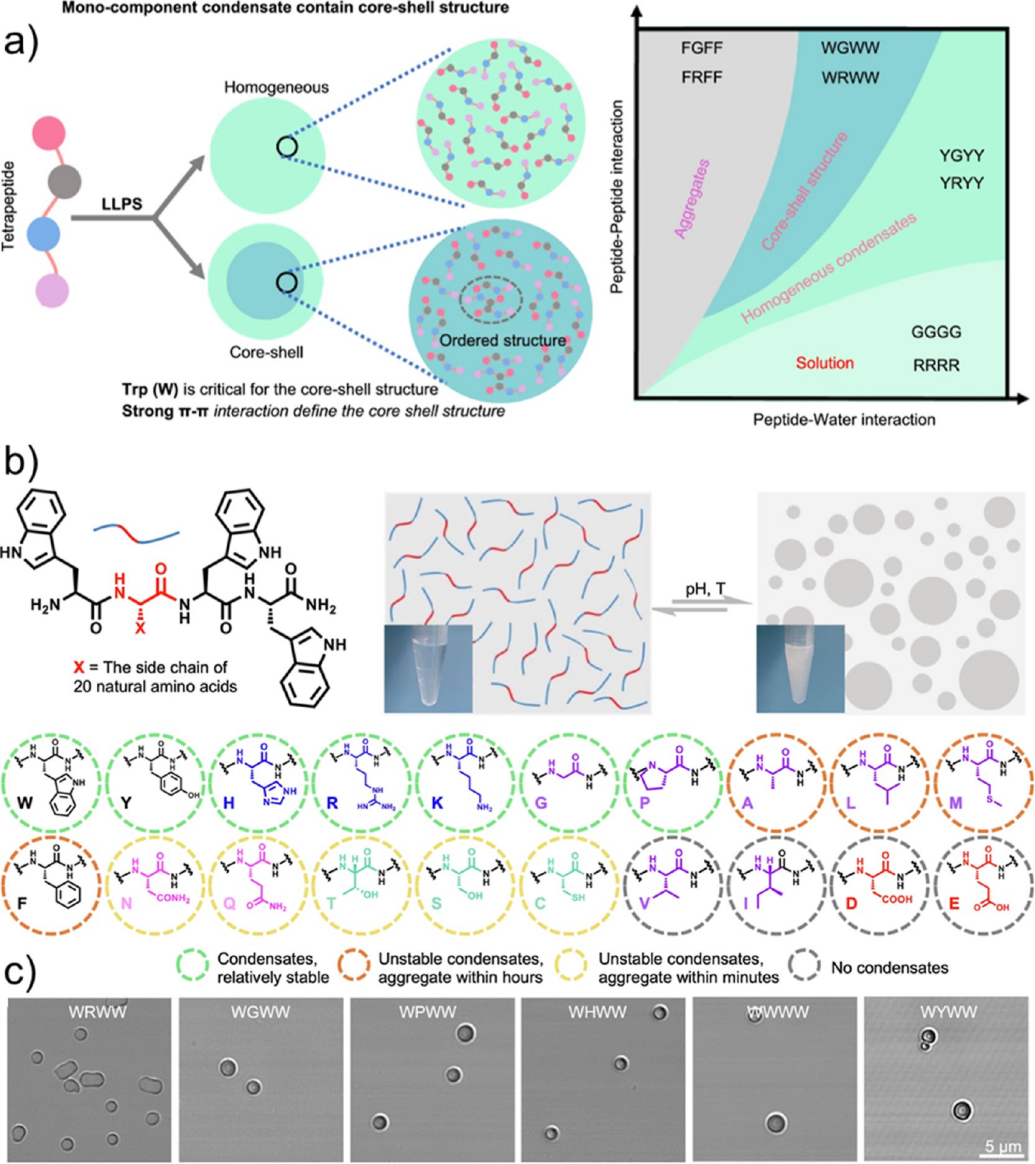
Schematic illustration, chemical design and phase diagram, (a) design representation and phase diagram, (b) model chemical structure and drawing of coacervates induced through pH and temperature (inset-picture of vials after and before coacervation), (c) optical microscope images. Reproduced from ref 63 available under a CC-BY-NC license, @ copyright 2025, Springer Nature.

Dipeptide stickers with disulfide spacers enable systematic evaluation of how amino acid identity and position in the sequence influence phase separation. In a representative study of homotypic systems, Zhang and co-workers examined a series of XX-ss-XX derivatives with dipeptides composed exclusively of nonpolar residues.^64^ Under alkaline conditions, where terminal amines are deprotonated and electrostatic effects are minimized, side chain–side chain interactions dominate as the primary driving force for phase separation. Importantly, a strong correlation between the phase-separation tendency and amino acid molecular mass was identified, underscoring the scaling of cohesive interactions with side-chain size.

Dipeptide stickers serve as minimalistic scaffolding components for coacervate formation, as demonstrated by several recent representative studies. Across these reports, the identity of the sticker is of paramount importance, with relative hydrophobicity emerging as a key determining factor. In a generalized dipeptide sticker format (XXssXX), the selection of the amino acid X requires consideration of two fundamental questions: (i) can X engage in noncovalent interactions with itself, and (ii) what is its degree of hydrophobicity? A systematic design approach typically begins with studying the corresponding two dipeptides (XXXX) in the absence of a spacer to evaluate its dominant self-assembly behavior, such as aggregation, coacervation, or gelation. Based on these insights, a spacer can then be introduced to modulate the phase behavior of the dipeptide system. Once a stable coacervate system is achieved, further tuning of amino acid identity enables the introduction of stimuli responsiveness, for example, through pH-sensitive terminal groups, redox-active spacers, or redox-induced cross-linking leading to membranization.

#### 3.1.2 Non-Natural Aromatic/Oligopeptides Motifs as Stickers

Oligopeptides are short chains of amino acids linked by amide bonds that have also been shown to self-assemble into nanostructures through noncovalent interactions.^65^ In the search for minimal peptide systems capable of undergoing liquid–liquid phase separation (LLPS) to form condensate droplets, Leshem et al. designed a library of minimalist peptides incorporating LLPS-promoting motifs.^66^ Building on this strategy, the model peptide incorporated aromatic residues (Trp and Tyr) together with Arg as an oligopeptide motif at one terminus. A nonpolar segment derived from the elastin-like polypeptide domain (VPGXG; where X represents any amino acid except proline) was positioned in the middle of the sequence to function as a spacer. This pentapeptide motif is widely used to engineer ordered/disordered polypeptides, and its hydrophobic residues are known to promote coacervation. A single aromatic residue was introduced at the opposite terminus, yielding the peptide sequence WGRGRGRGWPGVGY, termed as WGR-1. The cation–π and π–π stacking interactions are primary driving forces for self-coacervation. Although these peptides are longer than the minimal systems discussed earlier, the study clearly demonstrated that both the aromatic residue composition and the positioning of glycine-based spacers strongly influence the phase-separation propensity. Expanding on this work, the same research group developed a peptide library containing sequences with three, two, one, or no aromatic residues to systematically evaluate the role of aromatic motifs in driving coacervation with nucleotides. Importantly, the number of aromatic regions was found to directly regulate phase separation behavior, as well as the dynamics and stability of the resulting coacervates.^67^ Besides this, aromatic and cationic stickers could also tune the packing of molecules and diffusion in the coacervates, which could resultantly help to control the encapsulation efficiency of coacervates.^68^

Building on the established role of aromatic amino acids as sticker motifs, recent efforts to further simplify designer frameworks have focused on incorporating non-natural aromatic groups as alternative stickers (Figure 5a). In these systems, π–π interactions act as the primary driving force for phase separation, closely resembling the role of Fmoc aromatic groups in the self-assembly of amino acids and short peptides.^69,70^ In a study by Wang and co-workers, naphthalene was introduced as a non-natural aromatic sticker and ethylene glycol as spacer.^71^ The authors synthesized a Nap-o-Nap compound that undergoes LLPS in aqueous media to form coacervates upon the stoichiometric addition of cucurbit[7]­uril and adamantane via a cosolvent method, as shown in the microscopic images (Figure 5b,c). Interestingly, these coacervates exhibited dynamic fusion behavior, a key characteristic of protocell-like systems (Figure 5c). Furthermore, a pyrene aromatic motif was incorporated as a sticker in a related designer system.^72^ This pyrene-based compound similarly formed coacervates predominantly driven by π–π stacking interactions, as illustrated in the schematic representation and microscopic images (Figure 5d,e). A common theme between minimalistic natural or synthetic stickers is the incorporation of aromatic residues that promote pi–pi interactions as well as cation–pi interactions. Together, these studies demonstrate that fully synthetic aromatic motifs can effectively recapitulate the key physicochemical principles of phase separation while offering a simplified and tunable platform for engineering biomimetic compartments.

**Figure 5:**
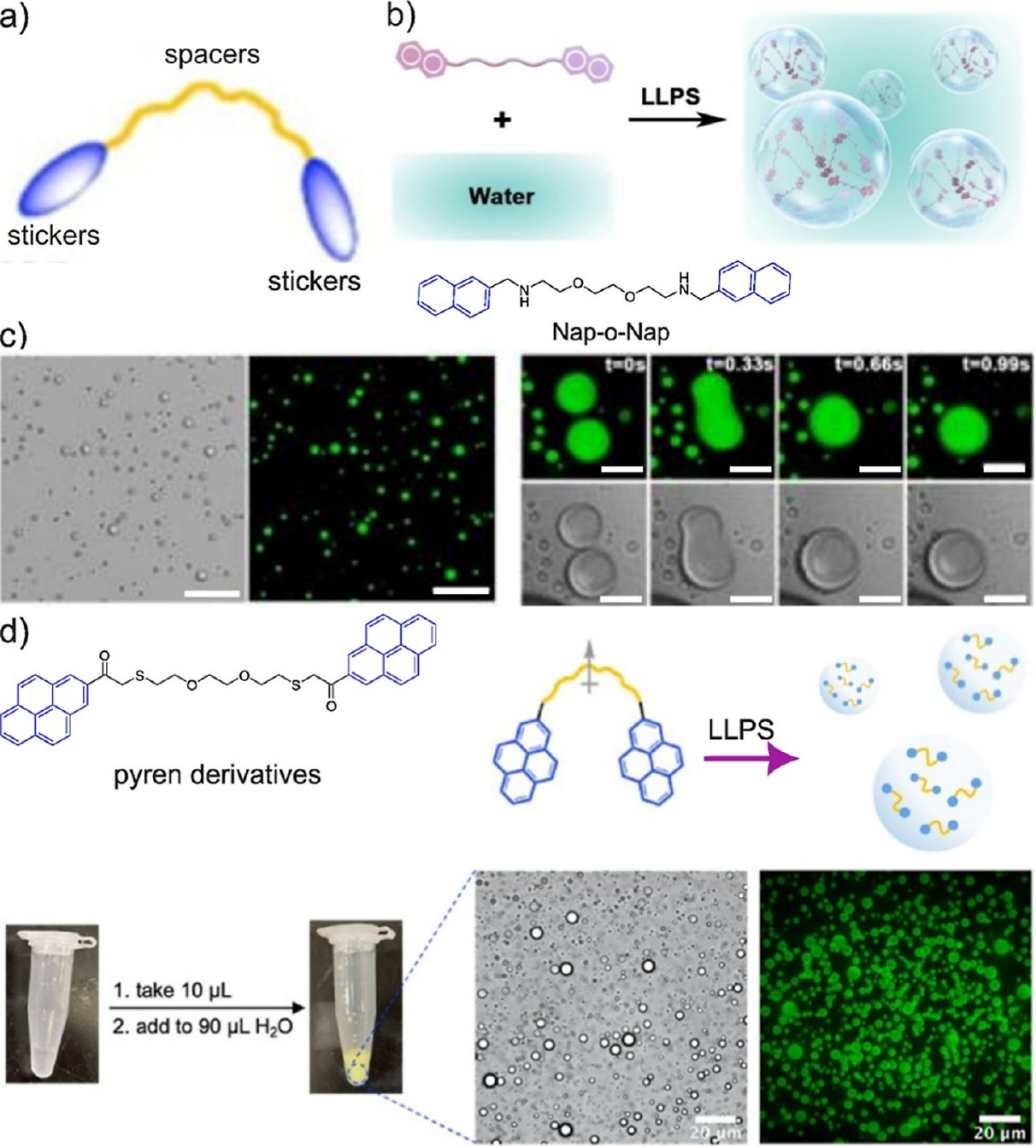
Schematic illustration to show the chemical design of sticker-spacer compound and phase separation, (a) sticker-spacer illustration, (b) chemical structure of Nap-o-Nap, (c) LLPS of Nap-o-Nap into coacervates and time series imaging of coacervates for fusibility. Scale bar −20 μm. Reproduced with permission from ref 71 @ Copyright 2025, John Wiley and Sons, (d) sticker-spacer-sticker where stickers are aromatic pyrene regions, (e) pictures of vials for inducing the LLPS, microscopic images of coacervates. Reproduced with permission from ref 72 @ Copyright 2023, John Wiley and Sons.

In this section, we highlight that sticker chemistry is the primary driver of LLPS, with aromatic and cationic residues (Phe, Tyr, Trp, and Arg) providing the multivalent interactions such as pi–pi stacking, cation–pi contacts, and hydrophobic forces needed for coacervate formation. A common trend suggests that too little aromaticity prevents phase separation, while too much promotes uncontrolled aggregation, meaning the sticker strength must be carefully balanced. Studies also reveal that the number, spacing, and positioning of sticker residues strongly influence the saturation concentration, droplet fluidity, and internal organization. Whether using minimal dipeptides, oligopeptides, or non-natural aromatic motifs, the core principle remains the same; that is, stickers impart interaction valency and dictate whether a system forms stable, dynamic droplets or collapses into irreversible solid-like aggregates.

### 3.2 Spacers for Coacervation

The linker motifs serve as spacers that can modulate the assembly pathway toward LLPS while also contributing to the overall chemical characteristics of the molecules that enable coacervate formation. In this section, we classify spacers into natural amino acids and non-natural linkers, and we further discuss the in situ generation of spacers through redox chemistry as an additional strategy to regulate the coacervation.

#### 3.2.1 Natural and Non-Natural Linkers as Spacers

The natural linkers are typically amino acids or short peptides that provide an appropriate balance between the apolar sticker and the polar spacer within the molecular design, thereby promoting LLPS and coacervate formation. The length of the spacer and its positional placement within the molecule are critical parameters for regulating the fluidity and dynamic behavior of the resulting coacervate droplets, for example, Ayala and co-workers reported a series of glycine-based spacers containing two to five residues.^68^ Interestingly, increasing the spacer length had a pronounced effect on the physicochemical properties of the resulting coacervates. The incorporation of glycine residues modulated charge density, polarity, and backbone flexibility, which in turn fine-tuned the dynamic behavior, molecular diffusion, and payload capacity of the corresponding sticker–spacer peptides. Given the importance of these linker strategies, we previously reported the use of non-natural linkers, such as cystamine, cadaverine, and others, as spacers with varying polarities within sticker–spacer frameworks. These synthetic linkers significantly altered the LLPS behavior, further demonstrating that precise control over spacer chemistry, whether natural or non-natural, is a powerful approach to modulate phase separation and coacervate properties.^46,62,73^

In one other study, Bao and co-workers designed a triphenylphosphine-based sticker–spacer model in which polyethylene glycol (PEG) chains of varying lengths (PEG_2_, PEG_4_, PEG_6_, and PEG_8_) were used to connect two triphenylphosphine groups.^74^ Phase separation was investigated in a DMSO/water solvent system, and the spacer length was found to play a decisive role in determining the assembly behavior. Compounds containing PEG_2_, PEG_4_, and PEG_6_ spacers exhibited aggregation, irregular droplets, and hollow droplets in aqueous solution, respectively, whereas the PEG_8_-linked compound formed well-defined coacervates. Similarly, Higashi and co-workers designed and synthesized a series of low-molecular-weight sticker–spacer compounds, abbreviated as OEG-bis-X, where X = NPmoc (4-nitrophenylmethoxycarbonyl), Pmoc (phenylmethoxycarbonyl), and cHex (cyclohexylmethoxycarbonyl).^75^ In this system, a flexible and hydrophilic oligoethylene glycol (OEG) chain served as the spacer, while the terminal X groups functioned as potential sticker units connected through carbamate linkages, and a combination of hydrogen bonding and hydrophobic interactions is the driving forces. The resulting assemblies exhibited stimuli responsiveness, droplet fusion behavior, and vesicular architectures with enhanced stability (Figure 6a). In addition to those vesicular structures, a strategy for multicompartmental architectures involved mixing a pair of core–shell coacervates, each containing a different fluorescent guest molecule. Intermixing of core fluorescence was achieved in this system due to the fast exchange of small and relatively hydrophilic guest molecules, while in another system the coacervates suppress intermixing because of the larger and more hydrophobic guest molecules. In a subsequent earlier study, the same group reported an OEG-based sticker–spacer system incorporating benzyl sulfide motifs as the sticker units.^76^ These compounds undergo coacervation, forming droplets that are redox-responsive. Specifically, the oxidation of the sulfide groups to sulfoxides disrupts the intermolecular interactions, leading to disassembly of the coacervates. Importantly, these assemblies can selectively sequester hydrophobic guest molecules, and their oxidative-triggered disassembly enables controlled release of encapsulated cargo (Figure 6b). Bao and co-workers also constructed a photoresponsive coacervate system in which fluorescent pyrene derivatives served as sticker motifs, connected via a photoactive carbonyl linker and a polyethylene glycol (PEG) spacer (see Section 3.3).^72^ It is therefore apparent that although the sticker-spacer model simplifies synthetic design of coacervate components, spacers and stickers chemistry mutually require careful selection as both components influence the resultant phase behavior under specific parameters. Nonetheless, these results further illustrate how precise modulation of spacer length and chemistry, whether natural or fully synthetic, provides a powerful handle to controlling LLPS and the resulting mesoscale structures.

**Figure 6:**
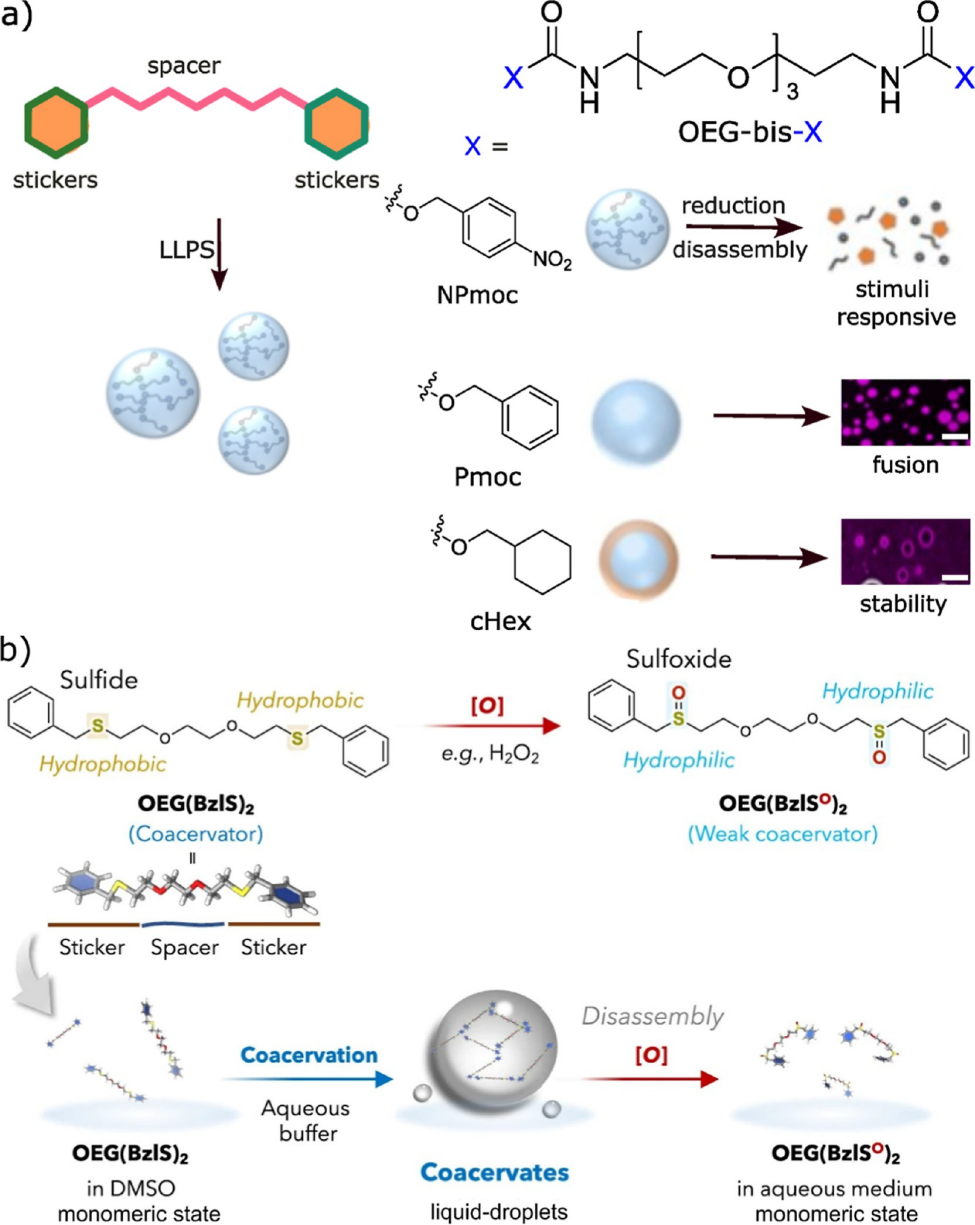
Schematic illustration of a synthetic sticker–spacer–sticker compounds, (a) three different sticker units were incorporated while maintaining the same oligoethylene glycol (OEG) spacer. Variation in the sticker chemistry resulted in distinct coacervate characteristics. Scale bar −5 μm. Reproduced with permission from ref 75 @ Copyright 2026, American Chemical Society, (b) sulfide benzyl as stickers and OEG as spacer framework shows the perfect control on assembly through oxidative disassembly of coacervates for controlled release of molecules. Reproduced from ref 76 available under a CC-BY license (The authors), @ Copyright 2025, Springer Nature.

#### 3.2.2 In Situ Formation of Spacers for Coacervation

Rather than relying on presynthesized natural or non-natural spacers, coacervation can also be achieved through the in situ formation of spacer units. A particularly effective strategy involves oxidation of thiol-containing sticker motifs or cysteine amino acid residue, which promotes covalent coupling between building blocks. This reaction-driven approach transforms otherwise static molecular systems into chemically adaptive assemblies, where phase behavior is dynamically regulated by ongoing redox processes.

Several recent studies highlight the potential of this concept, where reversible oxidative coupling and reductive decoupling have been used to control cycles of phase separation and dissolution. In one representative study, a highly water-soluble tetrapeptide, initially incapable of undergoing LLPS, was induced to phase separate upon oxidation of its thiol groups.^63^ Formation of disulfide bonds led to peptide dimerization (or oligomerization), effectively increasing the molecular length and valency. The resulting longer peptide species exhibited a significantly reduced saturation concentration and readily formed stable coacervate droplets. Importantly, the newly formed disulfide linkages function as dynamically generated spacer motifs within the sticker–spacer framework. By extension of the molecular architecture and modulation of intermolecular interactions, these covalent connections enhance droplet stability and reduce susceptibility to coalescence. Because disulfide bonds are redox-responsive, the assemblies can be reversibly dissolved upon reduction, enabling controllable cycles of assembly and disassembly.

Building on this concept, Wang and colleagues designed short cysteine-containing peptide sequences in which oxidative disulfide formation trigger the LLPS and lead to the formation of coacervate protocells.^77,78^ Besides materials design, such systems offer mechanistic insight into how subtle changes in molecular composition and connectivity influence phase behavior, with broader implications for understanding mutation-driven dysfunction in biological condensates (Figure 7a). These findings underscore the deterministic contribution of dynamic covalent spacer formation to regulating condensate stability. Similarly, Mondal and co-workers explored in situ disulfide bond formation using a library of cysteine-containing sticker–spacer peptides with varied sequence compositions.^79^ Across the library, the presence of cysteine consistently lowered the threshold concentration required for phase separation. The chemical capping of thiol groups suppressed the droplet formation, which confirms that redox-driven disulfide generation plays a central role in inducing coacervation (Figure 7b). Such in situ reaction-driven coacervation has also been demonstrated in non-natural stickers, and it is not limited to only redox reaction-mediated disulfide bond formation which could serve as spacer, but different types of reactions can form different spacers. For example, Patra and co-workers reported a boronic ester-based coacervate platform in which dynamic covalent interactions function as transient spacers within a sticker–spacer architecture.^80^ The reversible formation and hydrolysis of boronic esters enabled coacervates to assemble and disassemble in response to environmental cues, highlighting the adaptability of chemically driven coacervates. In another study, Xie and co-workers reported a sticker–spacer framework in which phenyl, carboxyphenyl, and naphthalene groups were employed as sticker motifs, while a cross-linking domain served as the spacer.^81^ By systematically tuning the hydrophobicity of the sticker units, they demonstrated distinct differences in the phase separation propensity, ultimately leading to the formation of coacervates. This reaction-mediated strategy provides a tunable, sequence-dependent route for coacervate formation.

**Figure 7:**
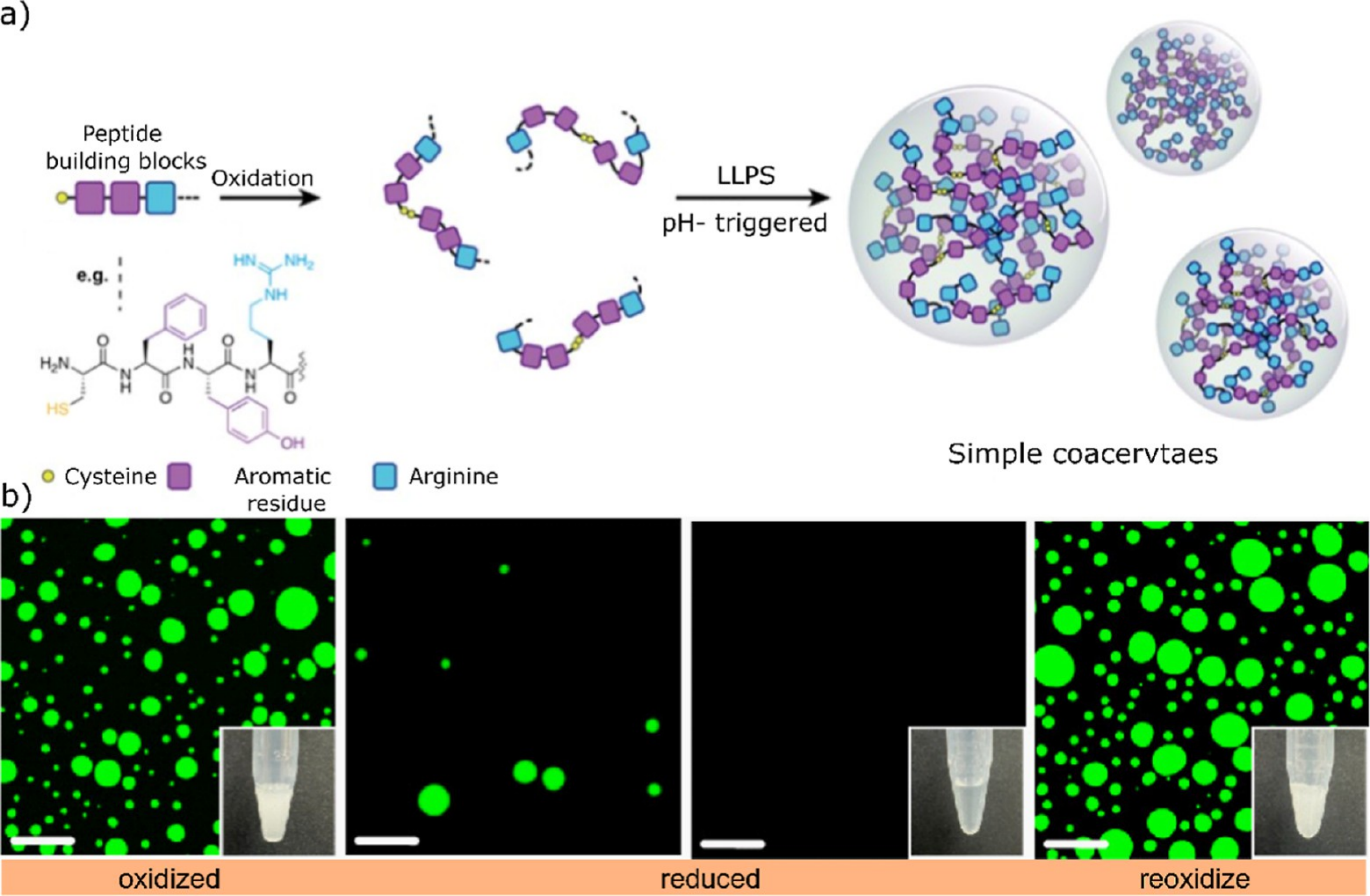
Schematic illustration stickers containing cysteine, (a) chemical design of cysteine containing peptides that undergoes oxidation to form spacers which formed coacervates through LLPS induced by pH, reproduced with permission from ref 77 @copyright 2025, Elsevier, (b) microscopic images after oxidation and reduction of disulfide bond and reformation, scale bar −20 μm. Reproduced from ref 79 available under license CC-BY-4.0 @copyright 2024, American Chemical Society.

These studies collectively highlight the versatility of sticker-spacer motifs, given the vast space of various chemistries and reactions. There is also an emphasis on the multifactorial nature of these sticker-spacer motifs, that while they are simplistic in essence, the interplay between sticker and spacer identity is what mainly governs coacervation and its subsequent stabilization. Hence, in this section, we have underscored how the chemical identity, length, and flexibility of spacers play a decisive role in tuning the phase behavior, stability, and functional responsiveness of sticker–spacer condensates. Across diverse systems, a consistent trend emerges; spacers modulate solubility, interaction valency, and the balance between fluid-like coacervates and solid-like aggregates. Hydrophilic or flexible spacers (cystamine, glycine-rich segments, or elastin-like motifs) generally promote reversible LLPS, enabling homogeneous mixing prior to phase separation and preventing premature aggregation. In contrast, less polar or shorter spacers increase sticker–sticker proximity, often driving nucleation into nanofibers or gels rather than stable droplets. Several studies demonstrate that spacer chemistry can encode stimuli-responsiveness such as redox-triggered transformations, pH-dependent assembly, or temperature-sensitive transitions. Importantly, systematic variation of spacer composition reveals that even minimal changes, such as introducing a single aromatic residue, altering spacer hydrophilicity, or modifying chain length, can significantly shift saturation concentrations, droplet morphology, and internal organization. These findings establish spacers as active phase separation regulators and show their central role in programming the dynamics and stability of sticker–spacer-based coacervates.

### 3.3 Light-Responsive Sticker-Spacer Compounds

Coacervate based protocells are stimuli-responsive biomaterials. Among the external stimuli capable of modulating coacervate behaviors, light has attracted particular interest due to its high spatial precision, rapid and reversible actuation, noninvasive nature, and facile tunability through wavelength and intensity control.^82,83^ The particularly appealing strategy involves the incorporation of photoactive moieties directly into coacervate building blocks, enabling direct spatiotemporal control over phase separation upon irradiation. In this context, sticker–spacer coacervates offer a highly versatile design framework, as demonstrated by several recent studies. For example, Bao and co-workers reported a photoresponsive coacervate system in which fluorescent pyrene derivatives served as sticker motifs, connected via a photoactive carbonyl linker and a polyethylene glycol (PEG) spacer.^72^ Fluorescence measurements revealed distinct emission intensities in the dilute and dense phases, confirming preferential partitioning of the pyrene moieties within the coacervates. Upon blue-light irradiation (405 nm), a photolytic Norrish reaction cleaved the carbonyl linker, triggering coacervate disassembly and a macroscopic transition from a turbid to a transparent solution. This photochemical response occurred efficiently on the time scale of minutes. The confocal fluorescence microscopy further demonstrated the cellular uptake of the coacervates, which was significantly reduced upon inhibition of energy-dependent endocytosis, which shows an active uptake mechanism. Importantly, these coacervates could encapsulate and deliver the functional protein cargo while preserving its biological activity within a cellular environment.

More recently, Kong and co-workers developed reversibly photoswitchable coacervates, formed using light responsive spiropyran sticker motifs separated by PEG spacers.^84^ Upon UV irradiation, spiropyran undergoes isomerization to its merocyanine form, which is both fluorescent and capable of generating reactive oxygen species (ROS) (Figure 8a,b). Subsequent exposure to visible light could reverse this transformation, that restore the nonfluorescent spiropyran state and resultantly suppress the ROS production. This reversible photoswitching represents a significant advantage over irreversible photoresponsive systems and offers a potential alternative to conventional photodynamic therapies, which often suffer from limited selectivity and prolonged cytotoxicity. The ROS generation by the merocyanine-based coacervates was confirmed by using singlet oxygen probes under visible-light irradiation, whereas the spiropyran state showed no detectable ROS activity. UV–vis spectroscopy further verified the reversible interconversion between the two photoisomeric states, underscoring the dynamic and controllable nature of this coacervate system.

**Figure 8:**
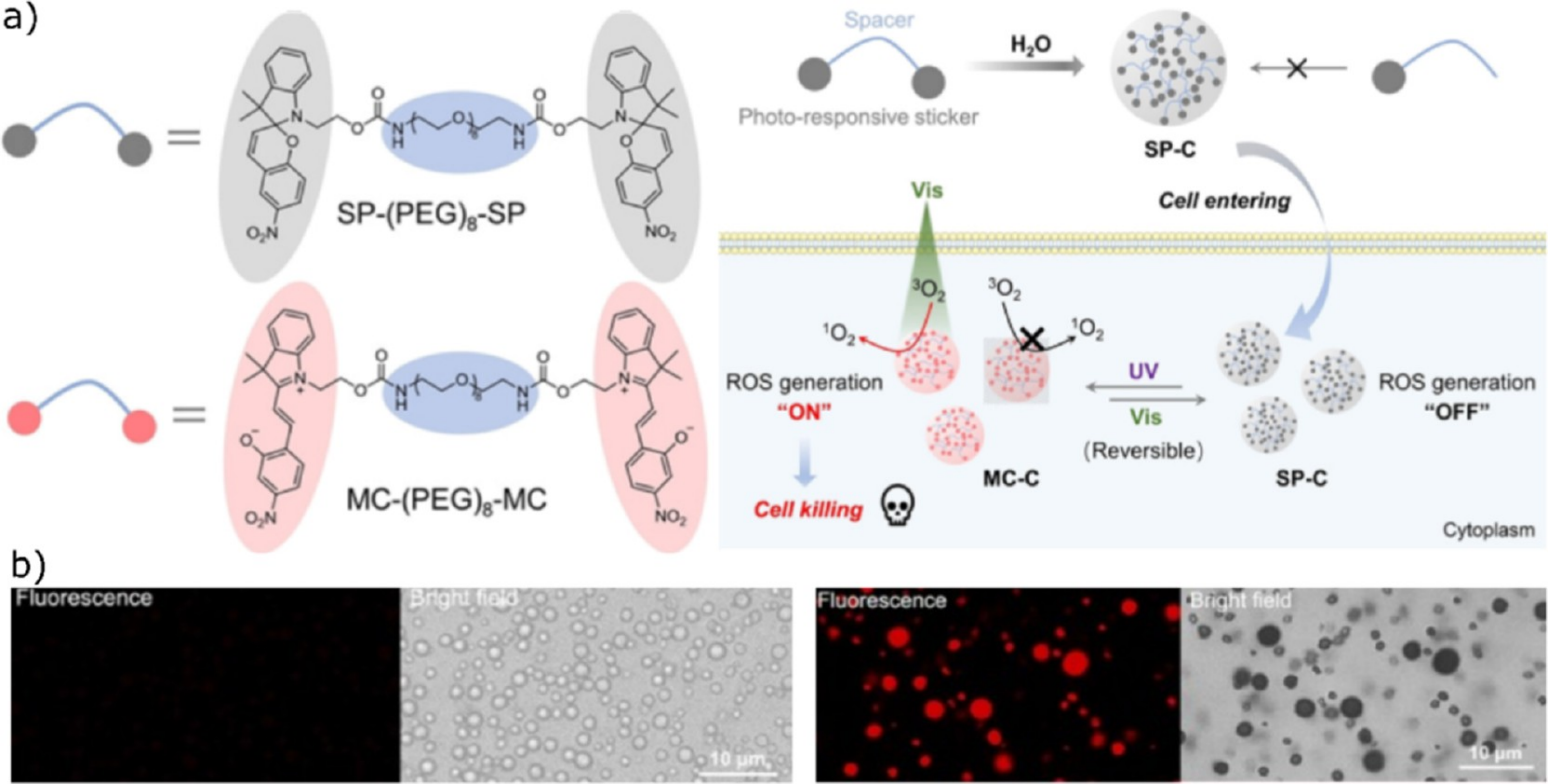
Chemical design and schematic illustration of coacervation, (a) chemical structures, coacervate formation, and ROS generation of fluorescent and nonfluorescent coacervates, (b) microscope images before and after isomerization. Reproduced with permission from ref 84 @Copyright 2025, John-Wiley and Sons.

### 3.4 Phase Transformation of Coacervates into Solid (Nanofibers)

The central objective in coacervate research is to identify systems that maintain prolonged stability before undergoing coalescence, droplet fusion, or irreversible aggregation. The stability of intermediate phases is particularly important in biological contexts, ranging from IDPs to mutagenic protein misfolding associated with pathological conditions. The systematic design of sticker–spacer frameworks provides a potent approach to uncover the molecular determinants governing phase separation and morphology. By varying amino acid sequences, polarity, and spacer length, researchers can directly correlate the molecular structure with material behavior. For example, we explored the effect of subtle sequence changes in sticker motifs by examining WFssFW, where a single amino acid substitution significantly altered the morphology and promoted aggregation, highlighting the strong impact of minor sequence modifications on material properties.^46^

In another study, solid-state NMR was employed to monitor liquid-to-solid transitions in sticker-spacer framework with aromatic dipeptides as stickers and cystamine as spacer, where π–π interactions are central to lead the phase transition.^60^ The time-resolved analysis revealed distinct transformation rates depending on the specific derivative. Complementary TEM imaging confirmed the formation of fibrillar structures with morphology strongly dependent on sticker-spacer framework composition (Figure 9). These findings underscore how small structural variations influence the pathway and kinetics of phase evolution. Interestingly, the spacer properties also play a decisive role in phase behavior; for example, the frameworks incorporating tyrosine-containing stickers with spacer to a five-carbon aliphatic chain that reduce spacer polarity dramatically altered assembly outcomes. While polar spacers favored coacervate formation, the more apolar C_5_ spacer rapidly underwent liquid-to-solid transition, often within less than 1 min^62^

**Figure 9:**
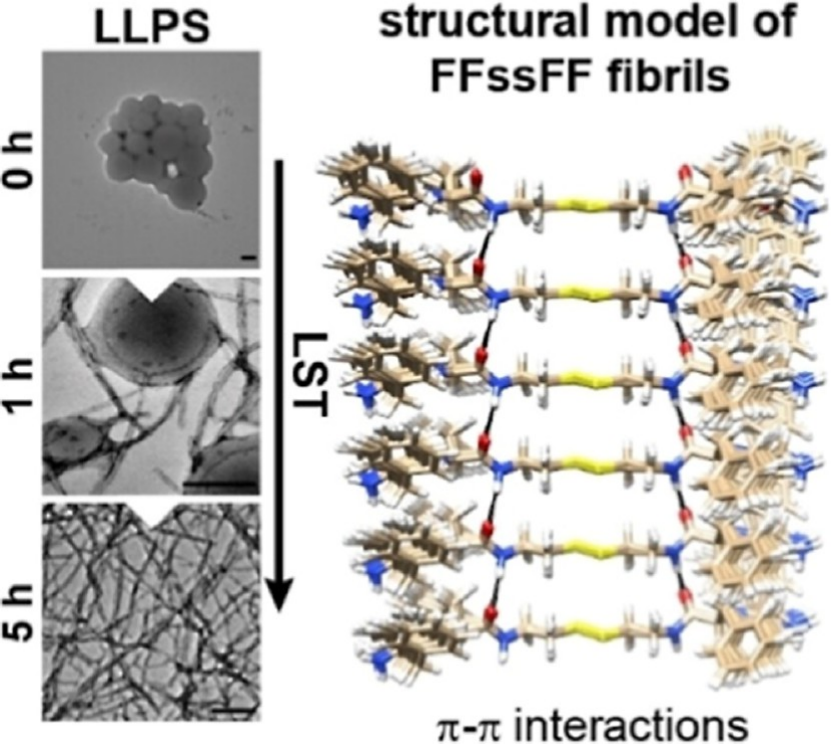
Liquid coacervates to solid transition (nanofibrils) of sticker-spacer frameworks, govern through π–π interactions of sticker regions. Scale bar −200 nm. Reproduced with permission from ref 60 available under CC-BY-NC license, @copyright 2023, John-Wiley and Sons.

Building on this concept, Younis et al. investigated an FF sticker motif linked via cadaverine instead of a conventional disulfide spacer which rather than forming coacervates, formed nanofibrous hydrogels, with assembly strongly influenced by buffer conditions and concentration of sticker-spacer framework.^73^ The increased hydrophobicity of the spacer likely played a significant role in accelerating the transition process and prevented the observable coacervate formation. A highly hydrophobic sticker spacer framework is subject to rapid formation of random aggregates, while a highly hydrophilic sticker spacer framework remains in the mixed solution state. In general, these studies demonstrate that achieving stable coacervates within the sticker–spacer framework requires a delicate balance between sticker and spacer polarity. Excessive hydrophobicity promotes rapid, arrested aggregation, whereas excessive hydrophilicity favors homogeneous mixing without phase separation. Therefore, the physicochemical properties of the sticker-spacer framework can be finely tuned through careful selection of amino acid identity, sequence, polarity, and spacer length to achieve controlled phase behavior and desired material states.

## 4 Applications of Sticker-Spacers-Based Coacervates

Over the past five years, coacervate-based membraneless protocells have emerged as distinctive biomaterials owing to their exceptional physicochemical properties, liquid-like behavior, and high capacity for loading client molecules.^85,86^ Among these systems, sticker–spacer-based coacervates are particularly promising for applications across diverse fields, including biocatalysis^46^ and macromolecular delivery.^77^ The membraneless coacervates enable rapid and reversible exchange of molecules with their surroundings while selectively sequestering guest species depending on their chemical nature. Moreover, their water-soluble formation allows high loading efficiencies without the use of organic solvents, thereby preserving the bioactivity of encapsulated components and enhancing both the catalytic performance and delivery efficiency. In the following sections, we focus on nonenzymatic reactions occurring within coacervates and examine their roles in the delivery of cargo macromolecules.

### 4.1 Chemical Reactions in Sticker-Spacer-Based Coacervates

One of the most intensively studied and compelling features of coacervates is their ability to locally concentrate reactants relative to the surrounding bulk phase.^24^ According to molecular collision theory, chemical reactions require reactants to encounter one another with an appropriate orientation in three-dimensional space. The sticker-spacer-based coacervate provide a confined and enriched environment in which long-range diffusion is no longer rate-limiting, leading to a remarkable enhanced frequency of molecular collisions.^9,10^ As a consequence, the effective activation energy associated with the transition state can be reduced, thereby accelerating the reaction kinetics. Owing to these properties, the coacervate phase is widely regarded as a sustainable and environmentally benign alternative to organic solvents traditionally employed for a broad range of chemical transformations.

Despite these advantages, effective reaction design within coacervates requires careful consideration of their chemical composition.^24^ Inadequate design can introduce functional groups that inhibit the reactions under investigation, as reported in several studies. In this regard, the sticker–spacer framework offers a powerful strategy to minimize synthetic complexity while enabling efficient catalysis across a wide range of reactions using a readily accessible library of modular peptide motifs. The environmental conditions under which coacervates form and persist are equally important, as external parameters such as temperature, pH, ionic strength, and periodic fuel consumption strongly influence droplet formation, stability, and dissolution. The effective local pH within coacervates can differ significantly from that of the bulk solution, and associated shifts in protonation equilibria may stabilize or destabilize specific reactants and products. This effect is especially pronounced in peptide-based coacervates containing ionizable side chains. By evaluating p*K*_a_ shifts, one can either identify reactions compatible with the coacervate microenvironment or rationally redesign the coacervate architecture to minimize undesired p*K*_a_ perturbations.

Besides contemporary reaction engineering, these properties have important implications for origin-of-life research. It has been proposed that primitive droplets formed and proliferated across prebiotic landscapes, where they were subjected to fluctuating environmental conditions, such as temperature changes. The persistence of such droplets implies an inherent ability to maintain structural integrity while supporting prebiotic chemistry and recursive self-reproduction.^66,87^ This hypothesis has motivated extensive investigation into coacervates as platforms capable of hosting nonenzymatic reactions without compromising coacervate stability, while simultaneously coupling product formation to further coacervation.

A notable example was reported by Wang and co-workers, who utilized ferricyanide and short lysine-rich peptides as coacervate building blocks.^88^ This system, proposed as a prebiotically relevant oxidizing environment, selectively sequestered metabolites, such as amino acids and α-amidothioacids, thereby promoting peptide bond formation within the coacervate phase. More recently, the same research group demonstrated enhanced reaction rates within coacervates formed via in situ spacer generation combined with a variety of sticker motifs.^77^ In this study, they investigated whether catalytically active amino acid residues could be incorporated into an LLPS-driven dimeric peptide design and evaluated the impact of phase separation on peptide catalysis (Figure 10a). Based on inspiration from natural hydrolases, which commonly feature a canonical Ser–His–Asp catalytic triad in their active sites, the authors focused on mimicking this motif, where the serine residue functions as the nucleophile that directly attacks substrates during hydrolysis reaction. In another study, the authors demonstrated lipase-mediated hydrolysis of 4-nitrophenyl hexanoate (4-NH) by encapsulating both the substrate and the enzyme within Nap-o-Nap coacervates.^71^ The reaction was monitored using UV–vis spectroscopy by tracking the formation of 4-nitrophenol. The reaction proceeded efficiently in the presence of coacervates but was significantly retarded upon their disassembly (Figure 10b).

**Figure 10:**
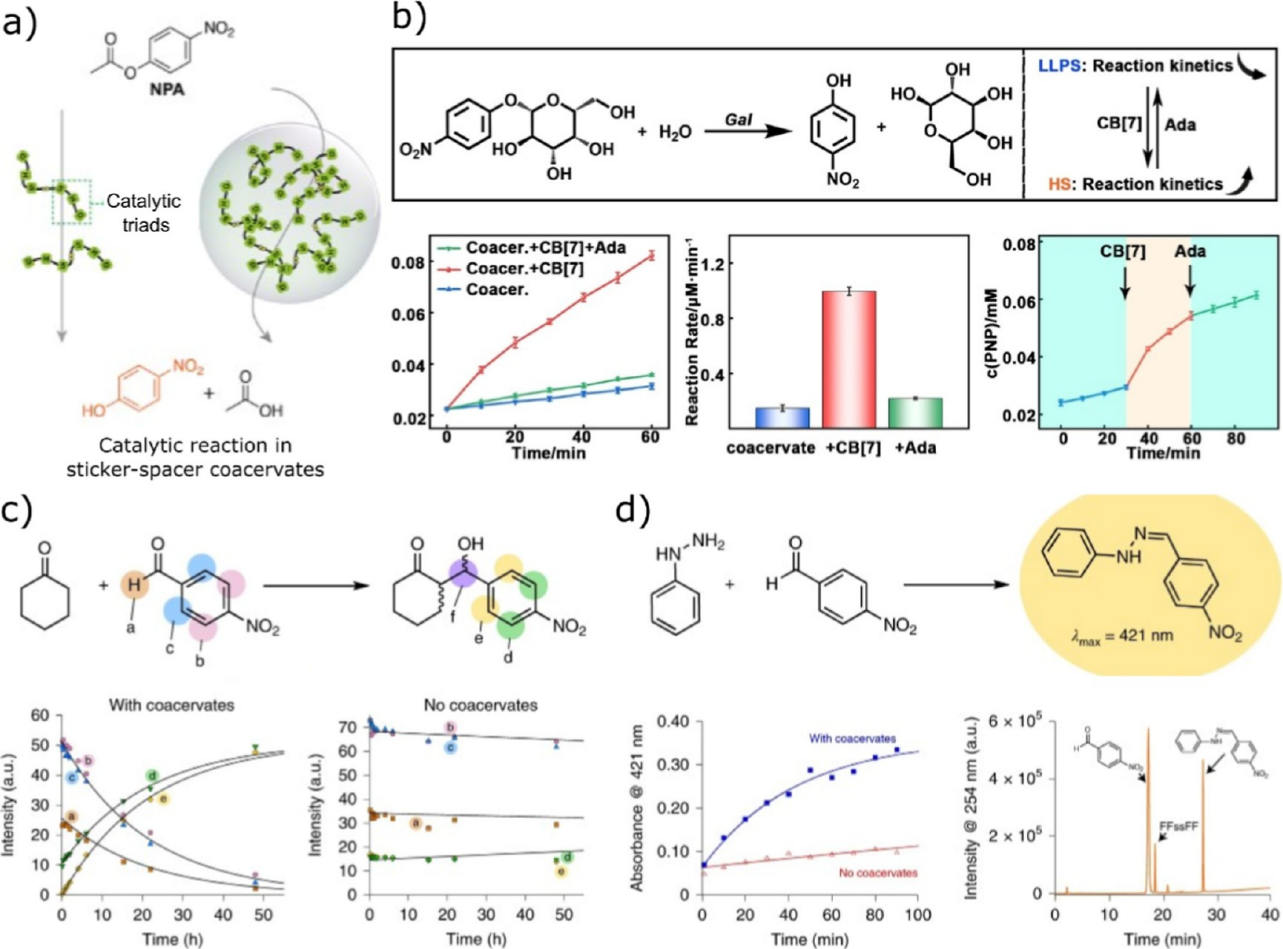
Chemical reaction schemes for catalytic activity inside sticker-spacer coacervates, (a) illustration of nonenzymatic reaction in the coacervates formed from in situ formation of spacers and then LLPS. Reproduced from ref 77 with permission from Elsevier, copyright 2025, (b) chemical scheme and rate enhancement in the presence of sticker-spacer coacervates. Reproduced from ref 71 with permission from Wiley-VCH, copyright 2025. (c,d) Chemical schemes for addition reactions inside the sticker-spacer coacervates and their rate enhancements. Reproduced from ref 46 with permission from Nature Publishing Group, copyright 2021.

The aldol addition reactions have been explored within simple coacervates formed from aromatic diphenylalanine motifs linked by a hydrophilic cystamine spacer.^46^ Using cyclohexanone and *p*-nitrobenzaldehyde as model reactants, the authors observed near-complete aldehyde consumption in the coacervate phase, while negligible conversion occurred in bulk solution over comparable time scales. Kinetic analysis revealed a pronounced rate enhancement, with the apparent reaction rate in coacervates exceeding that in bulk solution by more than an order of magnitude (Figure 10c). A similar improvement in the rate of reaction was also reported for hydrazone formation between phenylhydrazine and aldehydes, underscoring the versatility of coacervate-mediated rate enhancement for carbon–carbon and carbon–nitrogen bond-forming reactions (Figure 10d). Importantly, such reactivity is not restricted to simple sticker–spacer–based coacervates; similar enhancements have been demonstrated in more complex and multiphase coacervate systems, where reaction acceleration is often attributed to interfacial activation effects.^89,90^

Besides small-molecule conversion occurring within the coacervate interior, they are also capable of supporting the spatiotemporally regulated polymerization process. For example, Kubota and co-workers demonstrated that simple dipeptide-based coacervates can decode oscillatory light inputs, whereby light-triggered radical polymerization induces distinct, dynamic morphological changes within the coacervates, an effect reminiscent of signal transduction in biological systems.^91^ The encapsulation of monomers and initiators within the coacervate phase allowed polymerization to proceed selectively inside the droplets while remaining inactive in the surrounding bulk solution. Importantly, polymer formation was strongly dependent on the frequency of light irradiation, underscoring the ability of coacervates to temporally regulate reaction outcomes by modulating radical lifetimes in response to external stimuli. Owing to the unique and crowded microenvironment of coacervates, we describe sticker–spacer condensates as effective reaction crucibles in which a variety of chemical transformations can occur. The partitioning behavior of these membraneless compartments enables the efficient sequestration of a wide range of solutes, including prebiotically relevant molecules, as well as monomers and initiators for contemporary polymerization reactions.

### 4.2 Delivery of Drugs and Macromolecules

The recent surge in the design of both simple and complex coacervates has expanded their utility across a wide range of applications, particularly as carriers for nucleotide and drug delivery. Traditional delivery systems often suffer from low payload capacity, limited targeting specificity, and reliance on organic solvents during synthesis, which can lead to undesirable side effects.^92,93^ Polymeric complex coacervates may raise some concerns regarding immunogenicity as drug delivery platforms as they can trigger innate immune responses in the body. In contrast, minimalistic simple coacervates offer several advantages for macromolecule and drug delivery, including tunable size, high biocompatibility, and solvent-free formation.^94,95^

Beyond conceptual frameworks developed for low–molecular-weight coacervates, many of their intrinsic physicochemical properties are directly relevant to biomedical applications. Coacervates readily recruit molecules from their surroundings, resulting in high loading capacities, and have been shown to enhance the stability of therapeutics during circulation.^96^ Moreover, their stimuli-responsive behavior can be leveraged to achieve selective release in disease-associated microenvironments. For example, the acidic and glutathione-rich microenvironment of tumor tissues can trigger the disassembly of appropriately designed coacervates, enabling controlled therapeutic release.^26^

The chemical composition of coacervate forming sticker-spacers is a critical determinant of their function. For example, macromolecules such as polymer based coacervates are commonly employed due to strong interchain interactions, whereas low–molecular-weight coacervate frameworks offer simplified design strategies and serve as useful protocell models. Additionally, minimalistic peptide-based coacervates were shown to exhibit superior performance in protecting nucleic acid cargo when compared to their polymeric complex coacervate counterparts.^94^ Recent studies demonstrate that sticker–spacer-based coacervates can function as dynamic drug carriers that respond to disease-relevant stimuli. For instance, Chen and co-workers developed a redox-switchable peptide system in which tyrosine and histidine residues served as stickers linked by a disulfide bond.^59^ Under oxidative conditions catalyzed by horseradish peroxidase, these liquid-like coacervates transformed into semipermeable, membrane-bound structures, which reverted to a membraneless state under reducing conditions. Importantly, the particle size could be tuned during oxidation, a parameter known to influence circulation time and clearance in vivo. Nonoxidized coacervates exhibited more efficient cellular uptake, whereas oxidized structures showed enhanced circulatory stability. When complexed with siRNA, the oxidized coacervates significantly suppressed tumor growth in mouse models, an effect attributed to improved stability during circulation followed by reductive disassembly within the tumor microenvironment, enabling efficient intracellular delivery (Figure 11a–c).

**Figure 11:**
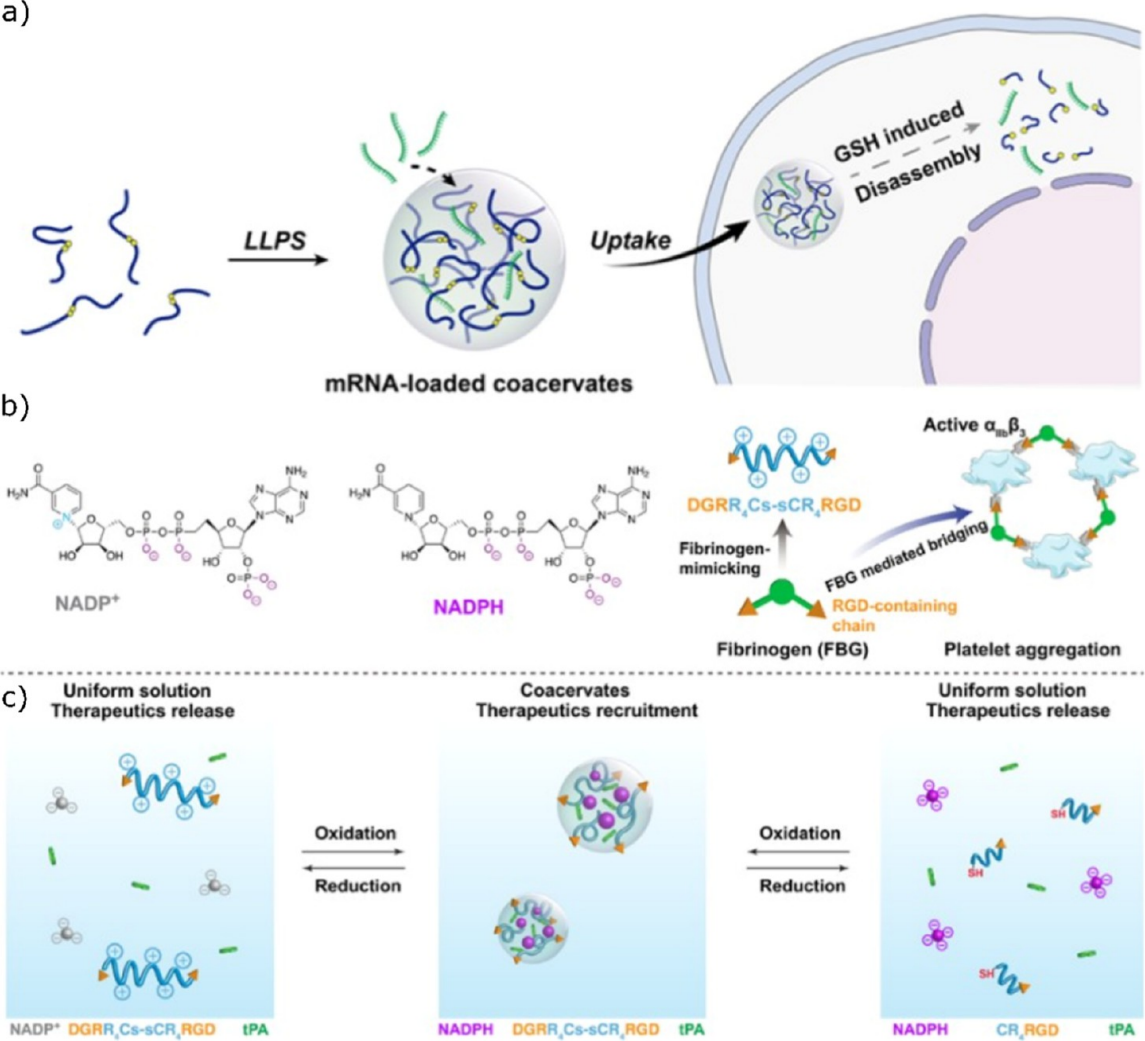
Schematic illustration of sticker-spacer frameworks for delivery of nucleotides and proteins, (a) design of sticker-spacer for LLPS mediated coacervate formation and cellular uptake. Reproduced with permission from ref 77 @copyright 2025, Elsevier, (b) chemical structure and schematic representation of fibrinogen mimicking sticker-spacer frameworks, (c) redox stimuli of coacervates for controlled release of cargo agents. Reproduced from ref 78 available under CC-BY-4.0 license, @copyright 2025, Springer Nature.

Building on redox-responsive delivery strategies, Wang and co-workers designed active complex coacervates composed of cationic arginine-containing peptides and negatively charged metabolites to encapsulate tissue plasminogen activator (tPA), a therapeutic protein used in acute ischemic stroke.^78^ Free tPA is rapidly cleared from circulation and can cause severe off-target effects, and encapsulation within peptide–metabolite coacervates enables the regulated release and enhanced thrombolytic efficacy. Targeting specificity was further improved by functionalizing the peptides with an RGD motif, allowing selective binding to activated platelets via αIIbβ3 integrins. These RGD-functionalized coacervates exhibited superior clot-lysing activity compared to nontargeted variants. The controlled release of tPA at clot sites was achieved through redox-triggered dissolution of the coacervates, highlighting the therapeutic potential of stimuli-responsive sticker–spacer frameworks.

Beyond small-molecule and nucleic acid delivery, coacervates are increasingly being explored for intracellular enzyme delivery, particularly for disorders involving an enzyme deficiency. Bao and co-workers demonstrated that β-galactosidase could be successfully delivered into the cytosol of HeLa cells using pyrene-based sticker–spacer coacervates.^72^ Following delivery, the enzymatic activity was confirmed using X-gal staining, indicating that the enzyme remained catalytically active after coacervate-mediated transport. This underscores the ability of peptide-based coacervates to deliver functional proteins without compromising their activity. Sticker–spacer coacervates have also been investigated as therapeutic agents. Kong and co-workers reported spiropyran-based coacervates that combine photoswitchable with photoinduced ROS generation.^84^ Upon light activation, these coacervates induced ROS-mediated cytotoxicity in multiple cancer cell lines. In-vivo studies further demonstrated selective tumor accumulation and significant tumor suppression upon light irradiation, with minimal systemic toxicity. Their results suggest the potential of photoresponsive coacervates for spatially controlled cancer therapy.

While targeting endogenous tumor biomarkers is a common therapeutic strategy, exploiting cellular stress-response machinery offers an alternative approach. SGs, which are membraneless organelles formed under cellular stress, play a critical role in cancer cell survival. Given the chronic stress conditions characteristic of tumor environments, disrupting SG function represents a promising anticancer strategy. Wang and co-workers developed a sticker–spacer sulfated tyrosine–phenylalanine peptide capable of undergoing in situ LLPS in response to lysosomal aryl sulfatase activity, a cancer-associated biomarker.^97^ Enzymatic conversion of the sticker-spacer peptide induces the coacervate formation within tumor cells, which selectively interfered with SG assembly through interactions with the RNA-binding protein G3BP2. These intracellular condensates significantly enhanced the efficacy of the anticancer drug sorafenib by promoting caspase-dependent apoptosis (Figure 12). In animal models, coadministration of sorafenib with the in situ forming coacervates remarkably decrease the tumor growth while maintaining favorable biosafety profiles. This strategy demonstrates how sticker–spacer based coacervates can be programmed to assemble selectively within tumor cells and disrupt stress-adaptive survival pathways.

**Figure 12:**
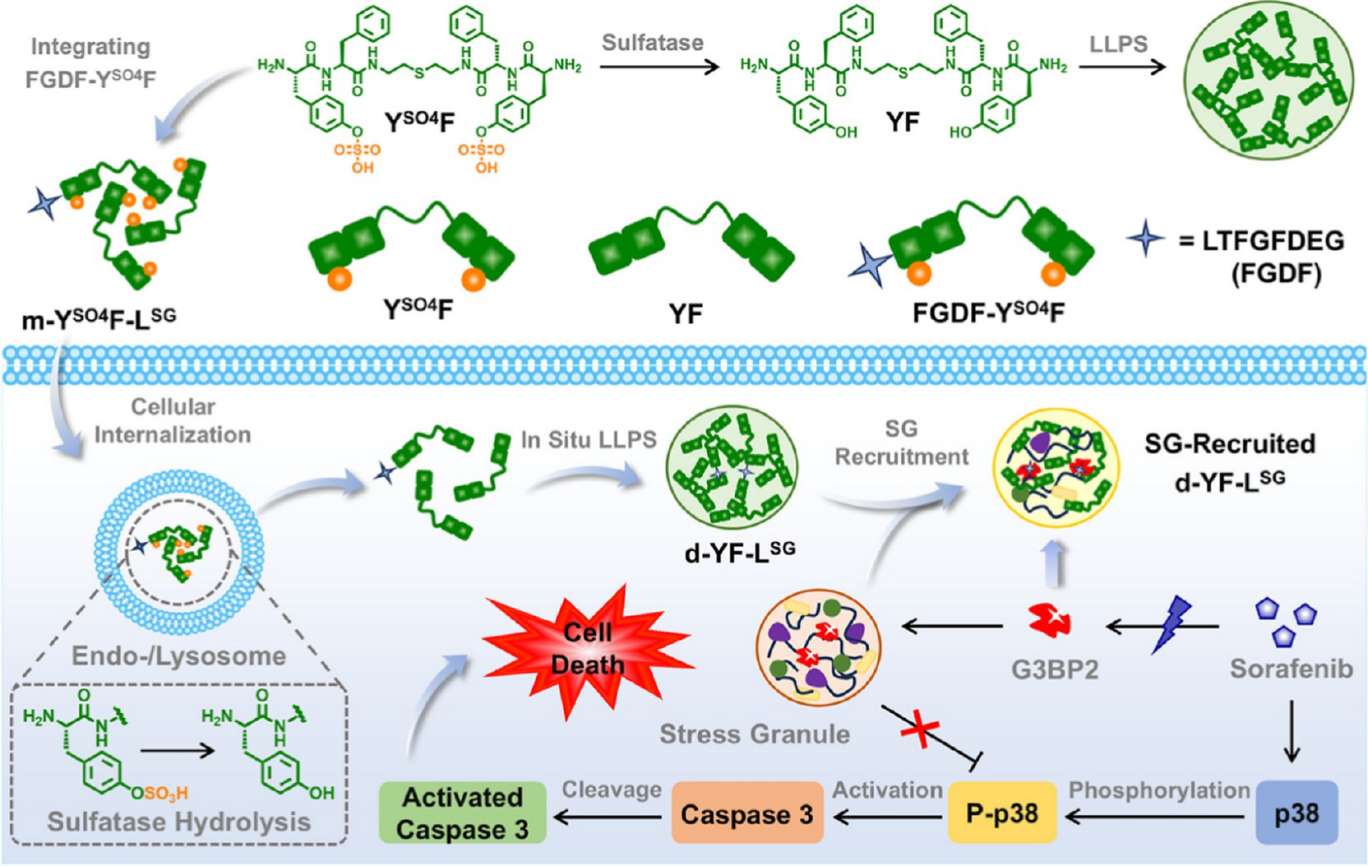
Schematic illustration to show the chemical design of sticker-spacer peptide derivative, structural transformation due to enzyme and in situ LLPS and mechanism of cell death along with cellular internalization. Reproduced with permission from ref 97 @ Copyright 2025, John Wiley and Sons.

In this section, we showed that sticker–spacer coacervates provide a potent and minimalistic platform for catalysis, compartmentalization, and therapeutic delivery by exploiting their ability to catalyze reactions and efficiently recruit diverse solutes. Their responsiveness to environmental cues, along with their compatibility with both prebiotic chemistry and modern biomedical applications, highlights their versatility. This positions these condensates as promising tools for accelerating reactions, protecting cargo, and enabling targeted, stimuli-controlled applications across chemical and biological systems.

## 5 Conclusions and Future Outlook

The sticker–spacer framework has evolved from a conceptual model for IDPs into a powerful design strategy for engineering small-molecule-based coacervate protocells with programmable properties. By delineating the roles of cohesive sticker motifs and solubilizing spacers, this approach enables precise molecular control that is often difficult to achieve in complex polymeric or biomacromolecule systems. As highlighted throughout this review, minimal building blocks, particularly peptides, exhibit remarkably diverse phase behaviors, including reversible LLPS, gelation, fibrillization, and chemically driven maturation. These transitions can be finely tuned through subtle modifications such as single-residue substitutions, spacer chemistry, or external stimuli, including light, pH, ionic strength, and temperature, underscoring the strength of reductionist design in elucidating the molecular basis of phase separation.

Beyond static phase behavior, dynamic processes such as in situ spacer formation, redox-mediated cross-linking, and photochemical switching demonstrate how coacervates can transition from passive condensates into adaptive, protocell-like systems. Such features not only provide insight into early biological compartmentalization but also position these materials as promising platforms for responsive biomaterials. Peptide-based sticker–spacer coacervates offer significant potential as microreactors, where compartmentalization can enhance reaction rates, concentrate reactants, and stabilize intermediates, especially in nonenzymatic systems that remain relatively underexplored.

Despite these advances, several challenges remain. Current studies predominantly focus on short peptide systems, leaving open questions regarding the applicability of sticker–spacer principles to larger, more complex biomolecules that better represent natural IDPs. While relationships between sticker valency, spacer properties, and phase behavior have begun to emerge, a comprehensive and predictive framework is still lacking. Additionally, critical aspects, such as long-term stability, biocompatibility, and scalability, require further systematic investigation, particularly for biomedical applications, such as drug and gene delivery.

Future research should prioritize the development of quantitative design maps that directly link molecular features to LLPS behavior, enabling predictive rather than empirical design. Expanding these systems to multicomponent and hierarchical coacervates represents an important next step toward mimicking the complexity of biological higher ordered life-like assemblies.^98^ Furthermore, incorporating a broader range of chemically responsive spacers capable of redox, pH, or light-triggered transformations will enhance functional versatility. In general, these efforts will advance sticker–spacer frameworks from minimal model systems to robust, programmable platforms with wide-ranging applications in materials science, chemistry, and biomedicine.

## References

[1] Oparin A. (1969). Chemistry
and the Origin of Life. R. Inst. Chem. Rev..

[2] Krishnamurthy, R. ; Hud, N. V. Introduction: Chemical Evolution and the Origins of Life; ACS Publications, 2020; Vol. 120, pp 4613–4615. 10.1021/acs.chemrev.0c00409. 32517476

[3] Delaye L., Lazcano A. (2005). Prebiological Evolution and the Physics
of the Origin
of Life. Phys. Life Rev..

[4] Peretó J. (2012). Out of Fuzzy
Chemistry: From Prebiotic Chemistry to Metabolic Networks. Chem. Soc. Rev..

[5] Yewdall N. A., Mason A. F., van Hest J. (2018). The Hallmarks of Living Systems:
Towards Creating Artificial Cells. Interface
Focus.

[6] Bar-Peled L., Kory N. (2022). Principles and Functions
of Metabolic Compartmentalization. Nat. Metab..

[7] Banani S. F., Lee H. O., Hyman A. A., Rosen M. K. (2017). Biomolecular Condensates:
Organizers of Cellular Biochemistry. Nat. Rev.
Mol. Cell Biol..

[8] Lyon A. S., Peeples W. B., Rosen M. K. (2021). A Framework for Understanding the
Functions of Biomolecular Condensates across Scales. Nat. Rev. Mol. Cell Biol..

[9] Smokers I. B., Visser B. S., Lipiński W. P., Nakashima K. K., Spruijt E. (2025). Phase-Separated Droplets Can Direct
the Kinetics of
Chemical Reactions Including Polymerization, Self-Replication and
Oscillating Networks. ChemSystemsChem.

[10] Smokers I. B., Visser B. S., Slootbeek A. D., Huck W. T., Spruijt E. (2024). How Droplets
Can. Accelerate Reactions– Coacervate Protocells as Catalytic
Microcompartments. Acc. Chem. Res..

[11] Podolsky K. A., Devaraj N. K. (2021). Synthesis of Lipid
Membranes for Artificial Cells. Nat. Rev. Chem..

[12] Martin N., Douliez J. (2021). Fatty Acid Vesicles
and Coacervates as Model Prebiotic
Protocells. ChemSystemsChem.

[13] Pir
Cakmak F., Marianelli A. M., Keating C. D. (2021). Phospholipid Membrane
Formation Templated by Coacervate Droplets. Langmuir.

[14] Maffeis V., Heuberger L., Nikoletić A., Schoenenberger C. A., Palivan C. G. (2024). Synthetic Cells Revisited: Artificial Cell Construction
Using Polymeric Building Blocks. Adv. Sci..

[15] Jiang W., Zhou Y., Yan D. (2015). Hyperbranched Polymer
Vesicles: From
Self-Assembly, Characterization, Mechanisms, and Properties to Applications. Chem. Soc. Rev..

[16] Fernandez-Trillo F., Grover L. M., Stephenson-Brown A., Harrison P., Mendes P. M. (2017). Vesicles
in Nature and the Laboratory: Elucidation of Their Biological Properties
and Synthesis of Increasingly Complex Synthetic Vesicles. Angew. Chem., Int. Ed..

[17] Gao N., Mann S. (2023). Membranized Coacervate Microdroplets: From Versatile Protocell Models
to Cytomimetic Materials. Acc. Chem. Res..

[18] Brangwynne C. P., Eckmann C. R., Courson D. S., Rybarska A., Hoege C., Gharakhani J., Jülicher F., Hyman A. A. (2009). Germline P Granules
Are Liquid Droplets That Localize by Controlled Dissolution/Condensation. Science.

[19] Feric M., Vaidya N., Harmon T. S., Mitrea D. M., Zhu L., Richardson T. M., Kriwacki R. W., Pappu R. V., Brangwynne C. P. (2016). Coexisting
Liquid Phases Underlie Nucleolar Subcompartments. Cell.

[20] Liu J., Zhorabek F., Chau Y. (2022). Biomaterial Design Inspired by Membraneless
Organelles. Matter.

[21] Uversky V. N. (2021). Recent
Developments in the Field of Intrinsically Disordered Proteins: Intrinsic
Disorder–Based Emergence in Cellular Biology in Light of the
Physiological and Pathological Liquid–Liquid Phase Transitions. Annu. Rev. Biophys..

[22] Dzuricky M., Rogers B. A., Shahid A., Cremer P. S., Chilkoti A. (2020). De Novo Engineering
of Intracellular Condensates Using Artificial Disordered Proteins. Nat. Chem..

[23] Alberti S., Gladfelter A., Mittag T. (2019). Considerations and
Challenges in
Studying Liquid-Liquid Phase Separation and Biomolecular Condensates. Cell.

[24] Harris R., Berman N., Lampel A. (2025). Coacervates
as Enzymatic Microreactors. Chem. Soc. Rev..

[25] Yuan J., Yang Y., Dai K., Fakhrullin R., Li H., Zhou P., Yuan C., Yan X. (2025). Peptide Coacervates:
Formation, Mechanism, and Biological Applications. ACS Appl. Mater. Interfaces.

[26] Souri M., Yim W., Halder M., Jin Z., Jokerst J. V. (2025). Coacervate-Based
Delivery Systems: Bridging Fundamentals and Applications. ACS Appl. Mater. Interfaces.

[27] Chattaraj A., Shakhnovich E. I. (2025). Separation of Sticker-Spacer Energetics
Governs the
Coalescence of Metastable Condensates. Biophys.
J..

[28] Abbas M., Lipiński W. P., Wang J., Spruijt E. (2021). Peptide-Based Coacervates
as Biomimetic Protocells. Chem. Soc. Rev..

[29] Sing C. E., Perry S. L. (2020). Recent Progress
in the Science of Complex Coacervation. Soft
Matter.

[30] Firdharini C., Yildiz I., AlNaqbi H., Abbas M. (2025). Minimal Designer Peptides
for Dynamic Homotypic Coacervate-Based Protocell Models. ChemSystemsChem.

[31] Lu T., Nakashima K. K., Spruijt E. (2021). Temperature-Responsive Peptide–Nucleotide
Coacervates. J. Phys. Chem. B.

[32] Tian Y., Hu Q., Sun Z., Yu Y., Li X., Tian T., Bi X., Li Y., Niu B., Zhang Z. (2024). Colon Targeting Ph-Responsive
Coacervate Microdroplets for Treatment of Ulcerative Colitis. Small.

[33] Sharma P., Patwal P. S., Kumar B. P. (2026). Ph-Responsive
Regulation of Multiphase
Coacervate Wetting Via Phase Selective Enrichment of Fatty Acids. Chem. Sci..

[34] Love C., Steinkühler J., Gonzales D. T., Yandrapalli N., Robinson T., Dimova R., Tang T. Y. D. (2020). Reversible Ph-Responsive
Coacervate Formation in Lipid Vesicles Activates Dormant Enzymatic
Reactions. Angew. Chem., Int. Ed..

[35] Duan G., Haase M. F., Stebe K. J., Lee D. (2018). One-Step Generation
of Salt-Responsive Polyelectrolyte Microcapsules Via Surfactant-Organized
Nanoscale Interfacial Complexation in Emulsions (So Nice). Langmuir.

[36] Reis D. Q., Pereira S., Ramos A. P., Pereira P. M., Morgado L., Calvário J., Henriques A. O., Serrano M., Pina A. S. (2024). Catalytic
Peptide-Based Coacervates for Enhanced Function through Structural
Organization and Substrate Specificity. Nat.
Commun..

[37] Yuan C., Li Q., Xing R., Li J., Yan X. (2023). Peptide Self-Assembly
through Liquid-Liquid Phase Separation. Chem.

[38] Cao S., Ivanov T., Heuer J., Ferguson C. T., Landfester K., Caire da Silva L. (2024). Dipeptide
Coacervates as Artificial Membraneless Organelles
for Bioorthogonal Catalysis. Nat. Commun..

[39] Cao S., Zhou P., Shen G., Ivanov T., Yan X., Landfester K., Caire da Silva L. (2025). Binary Peptide Coacervates as an
Active Model for Biomolecular Condensates. Nat.
Commun..

[40] Wei M.-T., Elbaum-Garfinkle S., Holehouse A. S., Chen C. C.-H., Feric M., Arnold C. B., Priestley R. D., Pappu R. V., Brangwynne C. P. (2017). Phase Behaviour
of Disordered Proteins Underlying Low Density and High Permeability
of Liquid Organelles. Nat. Chem..

[41] Molliex A., Temirov J., Lee J., Coughlin M., Kanagaraj A. P., Kim H. J., Mittag T., Taylor J. P. (2015). Phase Separation
by Low Complexity Domains Promotes Stress Granule Assembly and Drives
Pathological Fibrillization. Cell.

[42] Wang S.-H., Zheng T., Fawzi N. L. (2024). Structure
and Interactions of Prion-Like
Domains in Transcription Factor Efg1 Phase Separation. Biophys. J..

[43] do
Amaral M. J., Freire M. H. O., Almeida M. S., Pinheiro A. S., Cordeiro Y. (2023). Phase Separation of the Mammalian Prion Protein: Physiological
and Pathological Perspectives. J. Neurochem..

[44] Wang J., Choi J.-M., Holehouse A. S., Lee H. O., Zhang X., Jahnel M., Maharana S., Lemaitre R., Pozniakovsky A., Drechsel D. (2018). A Molecular
Grammar Governing the Driving Forces
for Phase Separation of Prion-Like Rna Binding Proteins. Cell.

[45] Martin E. W., Holehouse A. S., Peran I., Farag M., Incicco J. J., Bremer A., Grace C. R., Soranno A., Pappu R. V., Mittag T. (2020). Valence and Patterning of Aromatic Residues Determine
the Phase Behavior of Prion-Like Domains. Science.

[46] Abbas M., Lipiński W. P., Nakashima K. K., Huck W. T., Spruijt E. (2021). A Short Peptide
Synthon for Liquid–Liquid Phase Separation. Nat. Chem..

[47] Yan X., Zhu P., Li J. (2010). Self-Assembly and Application of
Diphenylalanine-Based
Nanostructures. Chem. Soc. Rev..

[48] Ruff K. M., Dar F., Pappu R. V. (2021). Ligand
Effects on Phase Separation of Multivalent Macromolecules. Proc. Natl. Acad. Sci. U.S.A..

[49] Yang Y., Jones H. B., Dao T. P., Castañeda C. A. (2019). Single
Amino Acid Substitutions in Stickers, but Not Spacers, Substantially
Alter Ubqln2 Phase Transitions and Dense Phase Material Properties. J. Phys. Chem. B.

[50] Naz M., Zhang L., Chen C., Yang S., Dou H., Mann S., Li J. (2024). Self-Assembly of Stabilized Droplets
from Liquid–Liquid Phase Separation for Higher-Order Structures
and Functions. Commun. Chem..

[51] Basavalingappa V., Bera S., Xue B., O’Donnell J., Guerin S., Cazade P.-A., Yuan H., Haq E. U., Silien C., Tao K. (2020). Diphenylalanine-Derivative
Peptide Assemblies with Increased Aromaticity Exhibit Metal-Like Rigidity
and High Piezoelectricity. ACS Nano.

[52] Diaferia C., Morelli G., Accardo A. (2019). Fmoc-Diphenylalanine
as a Suitable
Building Block for the Preparation of Hybrid Materials and Their Potential
Applications. J. Mater. Chem. B.

[53] Dignon G. L., Best R. B., Mittal J. (2020). Biomolecular
Phase Separation: From
Molecular Driving Forces to Macroscopic Properties. Annu. Rev. Phys. Chem..

[54] Li G., Yuan C., Yan X. (2025). Peptide-Mediated
Liquid–Liquid
Phase Separation and Biomolecular Condensates. Soft Matter.

[55] Chang R., Yuan C., Zhou P., Xing R., Yan X. (2024). Peptide Self-Assembly:
From Ordered to Disordered. Acc. Chem. Res..

[56] Sheehan F., Sementa D., Jain A., Kumar M., Tayarani-Najjaran M., Kroiss D., Ulijn R. V. (2021). Peptide-Based
Supramolecular Systems
Chemistry. Chem. Rev..

[57] Chibh S., Mishra J., Kour A., Chauhan V. S., Panda J. J. (2021). Recent
Advances in the Fabrication and Bio-Medical Applications of Self-Assembled
Dipeptide Nanostructures. Nanomed.

[58] Rissanou A. N., Georgilis E., Kasotakis E., Mitraki A., Harmandaris V. (2013). Effect of
Solvent on the Self-Assembly of Dialanine and Diphenylalanine Peptides. J. Phys. Chem. B.

[59] Chen R., Jiang Y., Lv M., Xu J., Liu Y., Qin J., Chen Z. S., Sun M., Fan Z., Du J. (2026). Redox-Regulated
Phase Switchable Peptide Droplets with Degradation Resistance for
Intravenous Delivery of Biopharmaceuticals. Adv. Mater..

[60] Lipiński W. P., Zehnder J., Abbas M., Güntert P., Spruijt E., Wiegand T. (2023). Fibrils Emerging from
Droplets: Molecular
Guiding Principles Behind Phase Transitions of a Short Peptide-Based
Condensate Studied by Solid-State Nmr. Chem.
Eur. J..

[61] Lampel A., McPhee S. A., Park H.-A., Scott G. G., Humagain S., Hekstra D. R., Yoo B., Frederix P. W., Li T.-D., Abzalimov R. R. (2017). Polymeric Peptide Pigments with Sequence-Encoded
Properties. Science.

[62] Abbas M., Law J. O., Grellscheid S. N., Huck W. T., Spruijt E. (2022). Peptide-Based
Coacervate-Core Vesicles with Semipermeable Membranes. Adv. Mater..

[63] Zhou L., Zhu L., Wang C., Xu T., Wang J., Zhang B., Zhang X., Wang H. (2025). Multiphasic
Condensates Formed with
Mono-Component of Tetrapeptides Via Phase Separation. Nat. Commun..

[64] Zhang Y., Prasad R., Su S., Lee D., Zhou H.-X. (2024). Amino Acid-Dependent
Phase Equilibrium and Material Properties of Tetrapeptide Condensates. Cell Rep. Phys. Sci..

[65] Kader S., Sultan M., Jabbari E. (2025). Self-Assembly of Homo Phenylalanine
Oligopeptides: Role of Oligopeptide Chain Length. Langmuir.

[66] Baruch
Leshem A., Sloan-Dennison S., Massarano T., Ben-David S., Graham D., Faulds K., Gottlieb H. E., Chill J. H., Lampel A. (2023). Biomolecular Condensates Formed by
Designer Minimalistic Peptides. Nat. Commun..

[67] Netzer A., Baruch Leshem A., Veretnik S., Edelstein I., Lampel A. (2024). Regulation of Peptide
Liquid–Liquid Phase Separation
by Aromatic Amino Acid Composition. Small.

[68] Veretnik S., Harris R., Lampel A. (2026). Glycine Composition
and Ion Valency
Tune Phase Behavior and Drug Encapsulation in Designer Peptide Condensates. ACS Appl. Mater. Interfaces.

[69] Smith A. M., Williams R. J., Tang C., Coppo P., Collins R. F., Turner M. L., Saiani A., Ulijn R. V. (2008). Fmoc-Diphenylalanine
Self Assembles to a Hydrogel Via a Novel Architecture Based on Π–Π
Interlocked Β-Sheets. Adv. Mater..

[70] Draper E. R., Morris K. L., Little M. A., Raeburn J., Colquhoun C., Cross E. R., McDonald T. O., Serpell L. C., Adams D. J. (2015). Hydrogels
Formed from Fmoc Amino Acids. CrystEngComm.

[71] Wang D., Zhou L., Zhang X., Zhou Z., Huang Z., Gao N. (2025). Supramolecular Switching
of Liquid-Liquid Phase Separation for Orchestrating
Enzyme Kinetics. Angew. Chem., Int. Ed..

[72] Bao Y., Chen H., Xu Z., Gao J., Jiang L., Xia J. (2023). Photo-Responsive Phase-Separating
Fluorescent Molecules for Intracellular
Protein Delivery. Angew. Chem., Int. Ed..

[73] Younis M., Tabish T. A., Firdharini C., Aslam M., Khair M., Anjum D. H., Yan X., Abbas M. (2025). Self-Assembled Peptide-Based
Fibrous Hydrogel as a Biological Catalytic Scaffold for Nitric Oxide
Generation and Encapsulation. ACS Appl. Mater.
Interfaces.

[74] Bao Y., Xu Z., Cheng K., Li X., Chen F., Yuan D., Zhang F., Che A. R.-Y., Zeng X., Zhao Y.-D. (2025). Staudinger
Reaction-Responsive Coacervates for Cytosolic Antibody Delivery and
Trim21-Mediated Protein Degradation. J. Am.
Chem. Soc..

[75] Higashi, S. L. ; Hirosawa, K. M. ; Fujimoto, R. ; Kanemaru, K. ; Yoshida, N. ; Suzuki, K. G. ; Ikeda, M. Sticker-Spacer Molecular Design Controls Coacervate Formation and Internal Microenvironments in low-molecular-weight Compounds; JACS Au, 2026. 10.1021/jacsau.5c01238 PMC12933334 41755842

[76] Fujimoto R., Higashi S. L., Shintani Y., Hirosawa K. M., Suzuki K. G., Ikeda M. (2025). Oxidation-Responsive
Coacervates Composed of Oligo (Ethylene Glycol)
Bearing Benzyl Sulfide Groups. Polym. J..

[77] Wang J., Abbas M., Qiu Y., Zhao Y., Wang J., Li Y. (2026). Sequence-Encoded Phase
Behavior and Functionality of Short Peptide
Coacervates. J. Colloid Interface Sci..

[78] Wang J., Abbas M., Huang Y., Wang J., Li Y. (2023). Redox-Responsive
Peptide-Based Complex Coacervates as Delivery Vehicles with Controlled
Release of Proteinous Drugs. Commun. Chem..

[79] Mondal M., Jankoski P. E., Lee L. D., Dinakarapandian D. M., Chiu T.-Y., Swetman W. S., Wu H., Paravastu A. K., Clemons T. D., Rangachari V. (2024). Reversible
Disulfide Bond Cross-Links
as Tunable Levers of Phase Separation in Designer Biomolecular Condensates. J. Am. Chem. Soc..

[80] Patra S., Sharma B., George S. J. (2025). Programmable Coacervate Droplets
Via Reaction-Coupled Liquid–Liquid Phase Separation (Llps)
and Competitive Inhibition. J. Am. Chem. Soc..

[81] Xie X., Li T., Ma L., Wu J., Qi Y., Yang B., Li Z., Yang Z., Zhang K., Chu Z. (2025). A Designer
Minimalistic Model Parallels the Phase-Separation-Mediated Assembly
and Biophysical Cues of Extracellular Matrix. Nat. Chem..

[82] Sawada D., Kojima T., Asakura K., Banno T. (2025). Light-Triggered Coacervation
of Low-Molecular-Weight Amphiphiles for Tunable Chemical Reactivity. Bull. Chem. Soc. Jpn..

[83] Martin N., Tian L., Spencer D., Coutable-Pennarun A., Anderson J. R., Mann S. (2019). Photoswitchable Phase Separation
and Oligonucleotide Trafficking in DNA Coacervate Microdroplets. Angew. Chem., Int. Ed..

[84] Kong H., Xie X., Bao Y., Zhang F., Bian L., Cheng K., Zhao Y. D., Xia J. (2025). Phase-Separated Spiropyran Coacervates
as Dual-Wavelength-Switchable Reactive Oxygen Generators. Angew. Chem., Int. Ed..

[85] Lu T., Javed S., Bonfio C., Spruijt E. (2023). Interfacing Coacervates
with Membranes: From Artificial Organelles and Hybrid Protocells to
Intracellular Delivery. Small Methods.

[86] Wang C., Shang L. (2025). Coacervate-Based Materials: Fabrication,
Structure, and Applications. Chem. Mater..

[87] Slootbeek A. D., van Haren M. H., Smokers I. B., Spruijt E. (2022). Growth, Replication
and Division Enable Evolution of Coacervate Protocells. Chem. Commun..

[88] Wang J., Abbas M., Wang J., Spruijt E. (2023). Selective Amide Bond
Formation in Redox-Active Coacervate Protocells. Nat. Commun..

[89] Peyraud-Vicré K., Dechamps C., Martin N., Desvergnes V. (2025). Coacervate
Droplets Drive Organocatalyzed Aqueous C–C Bond Formation Via
Interfacial Activation. J. Am. Chem. Soc..

[90] Dai Z., Meyn M., da Silva L. C. (2026). Organocatalytic
Peptide Coacervates
as Microreactors for Aqueous Aldol Additions. Chem. Commun..

[91] Kubota R., Torigoe S., Hamachi I. (2022). Temporal Stimulus
Patterns Drive
Differentiation of a Synthetic Dipeptide-Based Coacervate. J. Am. Chem. Soc..

[92] Manzari M. T., Shamay Y., Kiguchi H., Rosen N., Scaltriti M., Heller D. A. (2021). Targeted Drug Delivery Strategies for Precision Medicines. Nat. Rev. Mater..

[93] Johnston A. P., Such G. K., Ng S. L., Caruso F. (2011). Challenges
Facing Colloidal
Delivery Systems: From Synthesis to the Clinic. Curr. Opin. Colloid Interface Sci..

[94] Ding B., Jiang W., Ouyang T., Xu H. (2025). Smart Coacervate
Microdroplets:
Biomimetic Design, Material Innovations, and Emerging Applications
in Biomacromolecule Delivery. Bioact. Mater..

[95] Song S., Ivanov T., Yuan D., Wang J., da Silva L. C., Xie J., Cao S. (2024). Peptide-Based
Biomimetic Condensates Via Liquid–Liquid
Phase Separation as Biomedical Delivery Vehicles. Biomacromolecules.

[96] Liu J., Spruijt E., Miserez A., Langer R. (2023). Peptide-Based Liquid
Droplets as Emerging Delivery Vehicles. Nat.
Rev. Mater..

[97] Wang W., Wang H., Zhang Z., Liu X., Hu B., Tian F., Ye Z., Shi L., Yu Z. (2025). In Situ Liquid-Liquid
Phase Separation of Peptides into Droplets Targeting Membraneless
Organelles for Enhanced Cancer Chemotherapy. Adv. Mater..

[98] Abbas M. (2026). Coacervates
to Prototissues: Lifelike Assembly. Trends Chem..

